# On‐Demand Chemically Degradable Hydrogels for Biological Applications

**DOI:** 10.1002/cbic.202500956

**Published:** 2026-03-23

**Authors:** Xinyi Sheng, Justin Kim

**Affiliations:** ^1^ School of Chemistry and Biochemistry Georgia Institute of Technology Atlanta Georgia USA

**Keywords:** biocompatible, bioorthogonal, chemically induced, hydrogel, on‐demand degradable

## Abstract

Across various wound care applications, device interfaces, drug depots, and cell cultures, materials often require rapid and clean removal. On‐demand chemically induced degradable hydrogels fulfill this requirement through small‐molecule triggers that cleave covalent crosslinks or disrupt noncovalent interactions. Some of them readily accommodate therapeutic functions such as anti‐inflammatory or antioxidant payload delivery while maintaining desired material properties, including self‐healing, robust wet adhesion, cytocompatibility, and traceless dissolution. Chemical triggers provide a scalable and rapid dissolution method along with easy removal. In this review, we summarize gelation and degradation mechanisms, commonly used chemical triggers, representative biological applications, and degradation kinetics for both covalent and noncovalent disruption. The advantages and limitations in biocompatible and bioorthogonal approaches are discussed in detail, along with mechanistic development prospects and current clinical challenges for on‐demand chemically degradable hydrogels.

## Graphical abstract

This review explores the mechanisms and kinetics of chemically induced on‐demand degradation in hydrogels designed for painless dressing removal and traceless dissolution across various biological applications. Covalent bond cleavage and noncovalent disruption triggers are compared, highlighting opportunities for load‐bearing scaffolds with faster trigger response, strong biocompatibility, high bioorthogonality, minimal invasiveness, broad accessibility, and tunable degradability to meet diverse clinical needs.

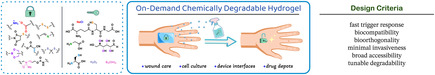

## 1 Introduction

Hydrogels, 3D polymer networks with high water content, low interfacial tension, and tunable porosity, exhibit extracellular matrix (ECM)‐like compliance and mass transport [1]. Natural polymers like collagen, gelatin, hyaluronan, chitosan (CS) and alginate, or synthetics, such as poly(vinyl alcohol) (PVA), polyacrylamide (PAM), poly(*N*‐isopropylacrylamide) (PNiPAAm), and poly(ethylene glycol) (PEG), have been used widely and tuned for hydrogel fabrication to achieve various adhesive strengths, gelation kinetics, mechanical properties, and degradation pathways [2]. Such biocompatibility and tunability enable hydrogels for tissue adhesives and interfaces for medical devices, wearables, hemostatic dressings, and regenerative platforms requiring intimate and durable contact with wet tissues [3].

**FIGURE 1:**
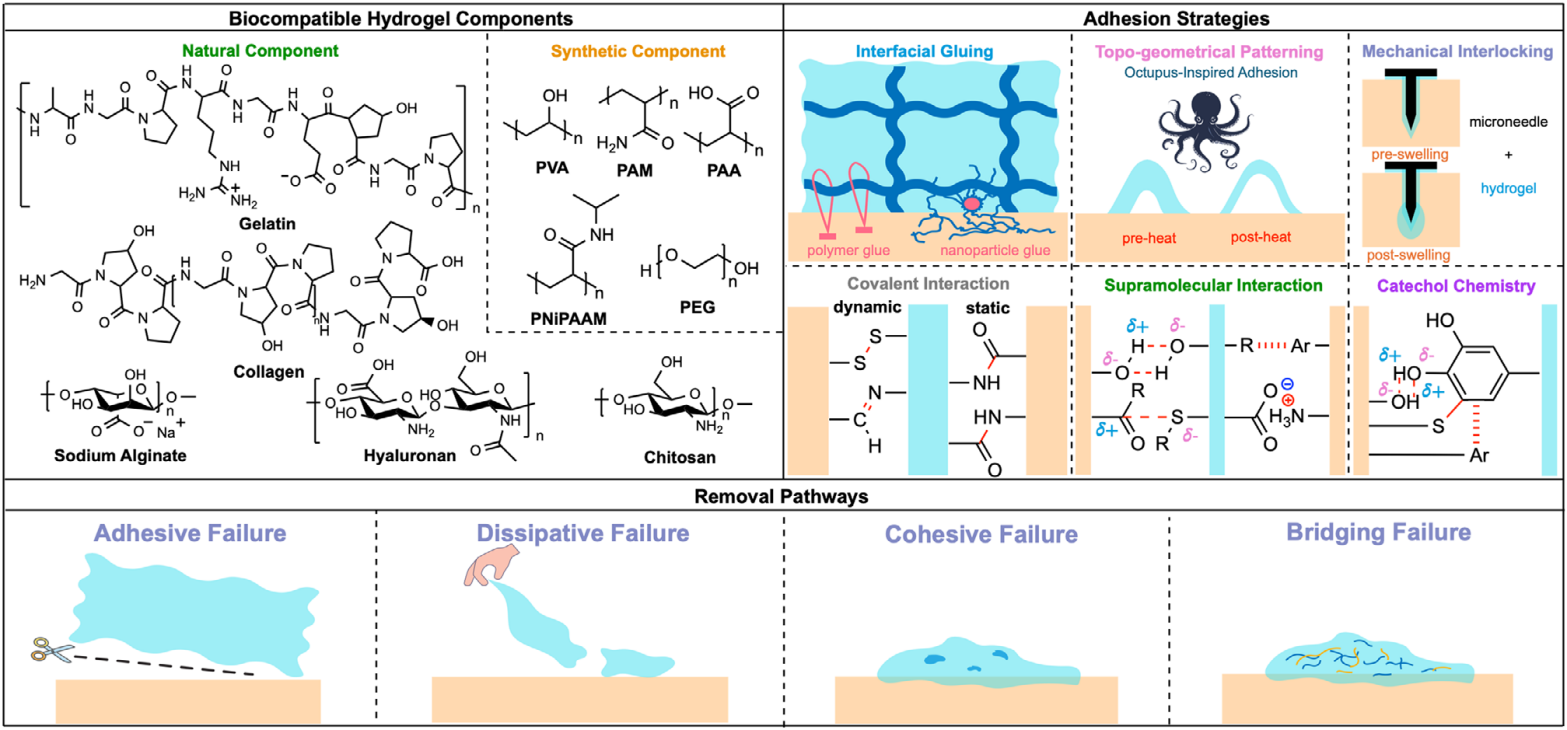
Schematic overview of chemical components, adhesion strategies, and removal pathways of tissue‐adhesive biocompatible hydrogels. Common natural and synthetic components used in biocompatible hydrogels are presented. Adhesive pathways include (1) nonchemical strategies: interfacial gluing using polymer or nanoparticle glues; topogeometrical patterning inspired by animal organisms such as octopus feet, in which the disc‐shaped feature can generate negative pressure between the gel and tissue surface after heating; and hydrogel‐coated microneedles that swell at the tip to achieve mechanical interlocking; and (2) chemical strategies: covalent interactions, supramolecular, and catechol chemistry that combines covalent and noncovalent interactions. Representative modes of removal include adhesive failure at the tissue interface, dissipative failure when external forces exceed the energy dissipation capacity of the gel, cohesive failure arising from insufficient internal crosslinking (dark blue areas), and bridging failure via scission of bridging polymers (yellow lines), which ultimately leads to gel degradation (blue lines).

To achieve strong and reliable adhesion strength, various strategies have been investigated, considering factors such as material selection, fabrication techniques, and the intended application. As depicted in Figure 1, physical/topographical routes include mechanical interlocking after swelling using microneedle coatings [4] and bioinspired surface architectures that generate negative pressure or capillary bridges [5], while “interfacial glues” use molecular [6], nanoparticle bridges [7, 8], or nanowhisker glue [9] to couple soft, hydrated interfaces. Chemical routes span static covalent bonds (e.g., amide formation via carbodiimide/NHS [10]) that enable maximal stability and dynamic covalent linkages (e.g., imines [11] and disulfides [12]) that enable self‐healing and stimulus response. Supramolecular assemblies and catechol chemistry also provide reversible and wet‐adhesive mechanisms, either through noncovalent interactions (H‐bonding [13], van der Waals [14], electrostatic [14], hydrophobic [13], host–guest interactions [15, 16, 17], etc.) or a combination of covalent (Schiff base, Michael addition) and noncovalent bonds (hydrogen bonds, π–π stacking, π‐cation electrostatic, metal coordination, hydrophobic interactions) [18] among catechols and different functional groups on the tissue surface.

Across clinical scenarios, removability is just as important as adhesion. Conventional dressing changes or device detachment can damage fragile neotissue, prolong inflammation, and increase pain [19, 20]; by contrast, on‐demand dissolvable hydrogels permit atraumatic removal once the material has served its purpose and also enable site‐specific release of payloads. Multiple mechanisms that have been applied to target degradation include (1) adhesive failure, which aims to disrupt interactions at the hydrogel‐tissue interface without fully dissolving the gel [21]. This mode preserves the gel body for retrieval but can be dangerous if interfacial scission is incomplete. (2) Dissipative failure, which leverages applied shear or peeling to exceed energy dissipation [22]. It is practical when an external force can be controlled, but it is less suitable for fragile tissues. (3) Cohesive failure, which impairs the cohesive strength of the bulk hydrogel [23]. (4) Bridging failure, which aims to reverse the phase transition process of stitch polymers to break the interlock [24] (Figure 1). Behind these mechanisms, the concept of bioorthogonality plays a significant role, as any method designed to form or remove the gel should ideally minimize its impact on biological systems. This principle is emphasized in this short review, which correlates chemical reactivity and selectivity with macroscopic biomaterial performance in living systems.

Depending on the applied materials and mechanisms for adhesion, gelation, and degradation, these removal pathways can either undergo noninducible or inducible degradation to serve different material purposes. For noninducible biodegradable networks, they erode under endogenous conditions (hydrolysis [25], enzymes [26], oxidation [27]) with half‐lives set at formulation. These materials have been applied successfully in diverse scenarios, including implantable hydrogel devices, tissue engineering, and drug‐delivery systems. On the other hand, inducibly degradable hydrogels are engineered to remain stable in use yet undergo rapid, externally commanded disassembly. The degradation mechanisms due to nonchemical stimuli such as light [28], enzymes [29], pH [30], temperature [31], gas [32], magnetothermal [33], and ultrasound [34, 35] have been widely reported. These nonchemical triggers can at times provide sub‐millimeter spatial control and near‐instant actuation without introducing exogenous small molecules, useful for patterned detachment around sutures, image‐guided scaffold remodeling, or device release in accessible sites, but they can be constrained by line‐of‐sight optics, energy delivery hardware, attenuation by blood/tissue, thermal/phototoxicity limits, and heterogeneous field distributions that risk residual adhesion. In contrast, chemically induced on‐demand removal relies on somewhat benign reagents (e.g., chelators [36], reductants [37], nucleophiles [38], competitive diols [39], or bioorthogonal molecules [40]) that diffuse into the network to cleave covalent crosslinks or disrupt noncovalent architectures, enabling programmable, dose‐dependent kinetics in light‐inaccessible, fluid‐immersed, amorphous, or geometrically occluded sites (e.g., intraoral patches [40], GI leak sealants [41], deep wound dressings [42]). Using chemical triggers is generally low‐cost, scalable, and has the potential for high orthogonality to tissues and payloads. However, there are current challenges, such as diffusion limits in thick or tough gels and the need to validate biocompatibility and clearance of both triggers and fragments.

An ideal removable gel should combine biocompatibility, bioorthogonality, rapid on‐demand removability, precise spatial control, stability, and scalability, while also enabling programmable load‐bearing release and achieving mechanical properties tailored to various clinical applications. In this review, we provide a reaction mechanism‐centered survey of chemically induced, on‐demand removal of hydrogels in biological contexts. The discussion is organized by two principal mechanisms: (1) noncovalent bond cleavage and (2) covalent disruption. For each chemical trigger, the mechanism, kinetics, and biological applications will be highlighted. This scope builds on recent work in hydrogel design and on‐demand detachment and is intended to complement broader surveys for chemically induced degradable hydrogel mechanisms.

## 2 Noncovalent Disruption

Major noncovalent interactions include van der Waals, hydrogen bonding, electrostatic, hydrophobic, host–guest interactions, and metal‐ligand coordination. Compared to covalent interactions that provide a high‐strength, long‐lived framework, these interactions are weaker and highly reversible, enabling rapid assembly and on‐demand disassembly but at the cost of lower long‐term stability and tighter dependence on the local medium. On‐demand chemical triggers include salts for Debye screening, H‐bond competitors, chelators, competitive guests, and benign redox agents, in addition to nonchemical triggers such as pH, light, and temperature shifts. In this section, we detail the mechanisms, triggers, kinetics, and application trade‐offs for four major classes—hydrogen‐bond disruption, electrostatic interaction disruption, host–guest complex displacement, and metal‐ligand coordination disruption.

### 2.1 Hydrogen Bond Disruption

**FIGURE 2:**
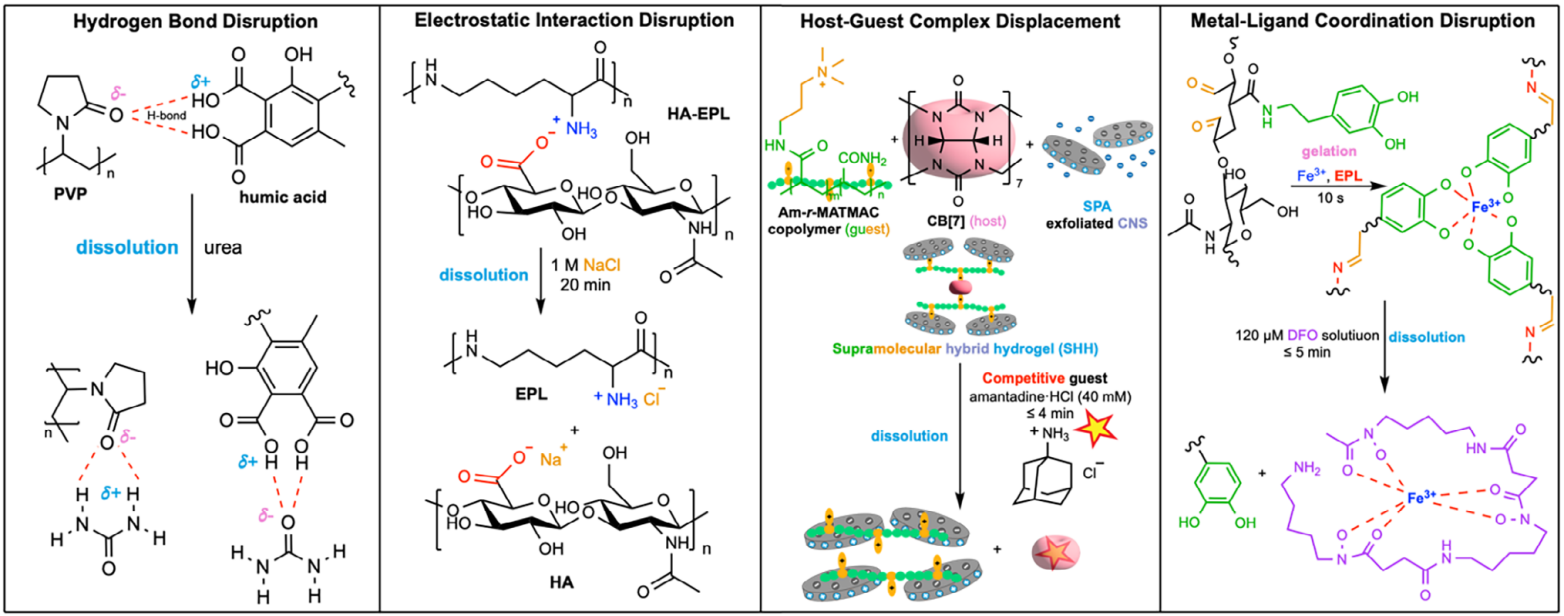
Representative examples of on‐demand chemically degradable hydrogels through noncovalent disruption. The hemostatic hydrogel formed using polyvinylpyrrolidone (PVP) and humic acid can be degraded by adding urea to disrupt the H‐bonds [45]. The injectable, self‐healing, wound dressing hydrogel HA‐EPL formed using hyaluronic acid (HA) and *ε*‐polylysine (EPL) can be degraded when treated with NaCl to disrupt the electrostatic interactions [47]. The burn‐dressing supramolecular hybrid hydrogel (SHH) formed using acrylamide‐random‐[3‐(methacryloylamino)propyl]trimethylammonium chloride (Am‐*r*‐MATMAC) copolymer, cucurbit[7]uril, exfoliated clay nanosheet (CNS), and sodium polyacrylate (SPA) can be degraded by adding competitive guest amantadine through host–guest complex displacement [48]. The in situ‐formed wound dressing hydrogel generated by mixing dopamine‐grafted oxidized HA, EPL, and FeCl_3_ can be dissolved via metal‐ligand coordination disruption by spraying deferoxamine mesylate (DFO) solution [49].

Hydrogen bonds are ubiquitous in biological systems and are desirable for biomaterials due to their moderate bond strength (2–251 kJ·mol^–1^) [43] and low intrinsic toxicity [44]. H‐bond disruption is one mode of hydrogel degradation that exists in the literature. Common functional groups employed in materials assembled through H‐bond networks include carboxyl‐hydroxyl, carboxyl‐amino, and carboxyl–carboxyl pairs [43], and disruption of these interactions is typically triggered by chaotropes like urea, polar solvent additives like DMSO and DMF, and sharp alterations to pH levels [45, 46]. Hydrogels designed around such strategies have been reported in applications targeting hemostasis. For example, a humic acid (HA)/poly(vinylpyrrolidone) (PVP) hydrogel forms within 10 s by mixing aqueous HA (6–10 wt%) and PVP (15 wt%). The robust mechanics and strong tissue adhesion arise from H‐bonds. Upon addition of urea, the interpolymer H‐bonds are disrupted, leading to hydrogel degradation (Figure 2). The gel also exhibits reversible thermo‐dissociation and self‐healing and is cytocompatible for hemostasis [45]. However, bioorthogonal methods to induce the degradation of H‐bonded networks do not seem to be available at this time.

### 2.2 Electrostatic Interaction Disruption

Electrostatic interactions arise from Coulombic interactions between charged ions and molecules within a medium. In hydrogels, common ion‐pair motifs are carboxylate‐ammonium and sulfate‐ammonium pairs characterized by ionic bond strengths ranging from 100 to 350 kJ·mol^–1^ [43]. The strength and lifetime depend on charge density, multivalency, and the surrounding ionic environment of the system. These interactions can be disrupted by altering the pH [50, 51] or increasing the ionic strength of the solution. The addition of salt weakens electrostatic interactions by Debye screening, and degradation usually occurs within minutes.

For example, Hu et al. reported an injectable, self‐healing polyelectrolyte complex wound dressing formed simply by mixing 4% (w/v) hyaluronic acid (HA) with 8% (w/v) *ε*‐polylysine (EPL) at a 10:3 (v/v) ratio, yielding shear‐thinning coacervate droplets with strong tissue adhesion for infected‐wound care. The network crosslinks arise from reversible electrostatic interactions, enabling rapid handling and conformal coverage. In vivo, the 8% HA‐EPL patch on rat skin was removed in 20 min using gauze soaked with 1 M NaCl (Figure 2) [47].

Electrostatic hydrogels assemble in water, respond rapidly and reversibly to changes in ionic content, and are easy to formulate from FDA‐familiar polymers such as alginate, CS, heparin, and common polycations. They are generally compatible with proteins and cells because no harsh chemistry is required. However, modest changes in ionic strength, pH, or protein content can erode mechanics and lifetime, undermining bioorthogonality and causing site‐to‐site variability in vivo. Applications of these methods are typically limited to external wounds or surfaces. Examples are not available for internal applications.

### 2.3 Host–Guest Complex Displacement

Host–guest interactions arise from the selective inclusion of a “guest” in a macrocyclic “host” cavity. Cyclodextrins (*α*‐, *β*‐, and *γ*‐CD) have hydrophilic exteriors and hydrophobic cavities. Cucurbiturils (CB[n]) form strong aqueous host–guest junctions, but they have a hydrophobic cavity lined with carbonyl‐containing, highly polar portals instead of a uniformly hydrophilic exterior. Other hosts include crown ethers, pillararenes, and calixarenes [52]. These ring‐shaped hosts rely on hydrophobic confinement, as well as cation‐π or ion–dipole interactions to facilitate binding [48, 53]. Reported host–guest bonds are typically in the 50–200 kJ·mol^–1^ range and are tunable by guest structure and medium [43]. The on‐demand disassembly occurs via guest exchange, where a guest with higher affinity competitively replaces the crosslinking guest in the host cavity (within 4 min using 40 mM amantadine·HCl solution, r.t.) [48], in addition to nonbiocompatible pathways such as redox‐triggered cleavage by converting hydrophobic ferrocene guest into a more hydrophilic ferrocenium species that is expelled from the hydrophobic host cavity (13 mM NaClO in borate buffer, pH 9, r.t.) [48, 54] and photo/thermo‐isomerization, where light or heat induces a *trans*→*cis* isomerization of azobenzene, weakening its inclusion in the host cavity so the guest is released and the network disintegrates (under UV irradiation at *λ* = 365 nm, r.t., or heat to 90°C) [55].

For example, Gokaltun et al. reported a self‐healing burn‐dressing hydrogel that forms in less than 1 min by mixing a cationic guest‐bearing copolymer with CB [7] and anionic, polyacrylate‐stabilized clay nanosheets (CNS), yielding an injectable network with strong tissue adherence. The crosslinks arise from hydrophobic and ion‐dipole recognition of host–guest pairs reinforced by electrostatic adhesion to the clay scaffold, enabling rapid handling and conformal coverage. On‐demand degradation is triggered by competitive guest exchange using amantadine·HCl (40 mM), and the bulk gel dissolves within 4 min (Figure 2) [48].

Host–guest supramolecular hydrogels can be tailored for injectability, self‐healing, and stimuli‐responsiveness in wound care, drug depots, and tissue engineering [52]. However, establishing quantitative correlations between structures and properties remains challenging. High local concentrations of competitors, oxidants, or light raise concerns about delivery and irritation. Media sensitivity to pH, ionic strength, and protein binding can soften gels. Trigger diffusion is also limited in thick construction. Other issues like leaching of host and guest species, limited host solubility, synthesis costs, and site‐to‐site variability of endogenous triggers further limit applications.

### 2.4 Metal‐Ligand Coordination Disruption

Metal‐ligand crosslinks arise from the coordination of Lewis basic ligands on a polymer (catechol [56], imidazole [57], carboxylate [58]) to metal cations (Fe^3+^/Fe^2+^, Zn^2+^, Ca^2+^, Mg^2+^, Cu^2+^) in solution [59]. The strength of these bonds spans a wide range from 8 to 439 kJ·mol^–1^ with metal‐ligand binding equilibrium constants ranging from 10^3^ to 10^40^ M^–1^ [43, 60]. Such reversible bonds form in water under mild conditions, making them appealing for bioadhesives, dressings, and injectable scaffolds. The effective lifetime is influenced by the metal's identity and valence, the ligand's denticity, the pH, which controls ligand protonation and metal speciation, and the presence of competing ions or ligands in the medium [59]. In addition to redox and pH stimulation, the coordination networks can be disrupted by chelators that outcompete the polymer ligand within minutes (e.g., dissolution within minutes using 25 mM EDTA, which is 4× excess of the polymer ligand His, to chelate Zn^2+^ [57]; dissolution within 50 min using 55 mM citrate, which is 10× excess of the polymer ligand alginate, to chelate Ca^2+^ [61]).

For example, Lv et al. engineered an in situ‐forming, multicrosslinked OD/EPL@Fe wound dressing hydrogel by mixing dopamine‐grafted oxidized HA (OD, 15 wt%) with EPL (20 wt%) and FeCl_3_ (3 mg·mL^–1^) at pH 8.5, yielding gelation and strong wet adhesion within 10 s. In seawater, gels swelled minimally and retained 33% mass at day 7, yet could be removed on demand by spraying 120 µM deferoxamine mesylate (DFO) solution within 5 min (Figure 2) [49].

Metal‐ligand coordination‐based hydrogels offer rapid, water‐based gelation with injectability and self‐healing, plus relatively strong noncovalent interactions within the system that can be disrupted by chelators. However, research has shown that degradation can happen through ion exchange for some metal coordination in biological systems [62]. Coordination crosslinks are also sensitive to pH change, and endogenous competing ligands include various primary metabolites, including amino acids and proteins, as well as essential ions such as phosphates, limiting their clinical applications.

## 3 Covalent Bond Cleavage

Covalent bonds exhibit greater strength compared to noncovalent interactions. Typical bond dissociation energies range roughly from 150 to 1000 kJ·mol^–1^ (C—C = 347, C—H = 413, C=O = 745 kJ·mol^–1^). In contrast, hydrogen bonds have energies of ≈8.4–41.8 kJ·mol^–1^ [63], π–π stacking has energies of roughly 4–63 kJ·mol^–1^ [64], and van der Waals contacts have energies of ≈4–8 kJ·mol^–1^ [65]. Consequently, covalent bonds often serve as the structural framework for polymer networks while noncovalent forces regulate the assembly of these structures. In the context of hydrogels, strong adhesion energy is especially important in applications like bone repair and post‐ostomy adhesives [2, 10]. In comparison to hydrogels that depend on noncovalent interactions and exhibit adhesion energies of ≈0.1−10 J·m^–2^, hydrogels that adhere via covalent linkages can achieve adhesion energies of over 1000 J·m^–2^ [66, 67]. For example, Mooney and coworkers reported a strong adhesive hydrogel to wet tissues through amide bond formation between amine‐rich CS and EDC/sulfo‐NHS‐activated carboxyl groups on tissue proteins. These hydrogels achieved adhesion energies up to 1116 J·m^–2^ on porcine skin [67].

To completely remove hydrogels from tissue, chemical methods that cleave covalent linkages at the adhesive interface (adhesive failure), within the network (cohesive failure), or at stitching molecules (bridging failure) are uniquely effective. Across on‐demand chemically degradable hydrogels, there are currently three major types of mechanisms, including (1) dynamic exchange by thiols, diols, amines, and retro‐Knoevenagel condensation; (2) ROS‐based cleavage; and (3) bioorthogonal dissociation. Due to the reversible nature of dynamic reactions, they are generally slow unless high concentrations of triggers are used, potentially undermining bioorthogonality. ROS‐sensitive hydrogels are not strictly bioorthogonal due to the presence of endogenous reductants and oxidants, such as glutathione and peroxides, respectively. Bioorthogonal dissociation offers the best compromise between degradation rate, reagent concentration, biocompatibility, and spatial and temporal precision.

### 3.1 Competitive Exchange

#### 3.1.1 Thiol‐Disulfide/Diselenide Exchange

Disulfides are generally stable under physiological conditions but can undergo rapid cleavage under reducing conditions in the presence of competitive thiols. Mechanistically, disulfide bonds undergo exchange with thiolates via nucleophilic exchange by an S_N_2 mechanism. This produces a new disulfide, integrating the new thiol while releasing one half of the original disulfide (Figure 3A). This rapid exchange is governed by an equilibrium process. Diselenides undergo a similar cleavage mechanism (Figure 3B), but with faster exchange rates due to a lower Se—Se bond energy (172 kJ·mol^–1^) compared to that of the S—S bond energy (240 kJ·mol^–1^) [70]. Cleavage triggers are generally low molecular weight thiols—most commonly glutathione (GSH) for endogenous applications and dithiothreitol (DTT) or cysteine for exogenous on‐demand removal. Hydrogels featuring these dichalcogenides exhibit tunable degradation times ranging from 10 min (0.16 M GSH in pH 7.4 PBS, r.t.) [68] to 5 h (0.1 M GSH in water, 60°C) [71] for disulfides and 1 min (10 mM GSH in water, r.t.) [69] for diselenides. This mechanism has been harnessed for developing chemically induced degradable hydrogels, where the cleavable disulfides or diselenides can be built into either the polymer backbone or side chains.

FIGURE 3
Schematic illustration of the thiol‐disulfide/diselenide exchange mechanism and examples of on‐demand chemically degradable hydrogels based on this principle. (A) Thiol‐disulfide exchange mechanism. (B) Thiol‐diselenide exchange mechanism. (C) A wound dressing hydrogel, formed by crosslinking thiol‐bearing 8‐arm‐poly(ethylene glycol) (8‐arm‐PEG‐SH) with either thiopyridyl‐terminated 8‐arm‐PEG (8‐arm‐PEG‐S‐TP) or H_2_O_2_ in phosphate buffer (PB), can be degraded upon exposure to glutathione (GSH) to form possible products 8‐arm‐PEG‐S‐SG, 8‐arm‐PEG(SH)‐S‐SG, 8‐arm‐PEG(SH)‐SH, and GSH disulfide via thiol‐disulfide exchange [68]. (D) A burn‐dressing hydrogel, formed by diselenide crosslinking of *γ*‐selenobutyrolactone‐modified chitosan, can be degraded when treated with GSH solution via thiol‐diselenide exchange [69]. Throughout the paper, functional groups responsible for degradation are colored in red, while chemical triggers are colored in blue.

**Figure:**
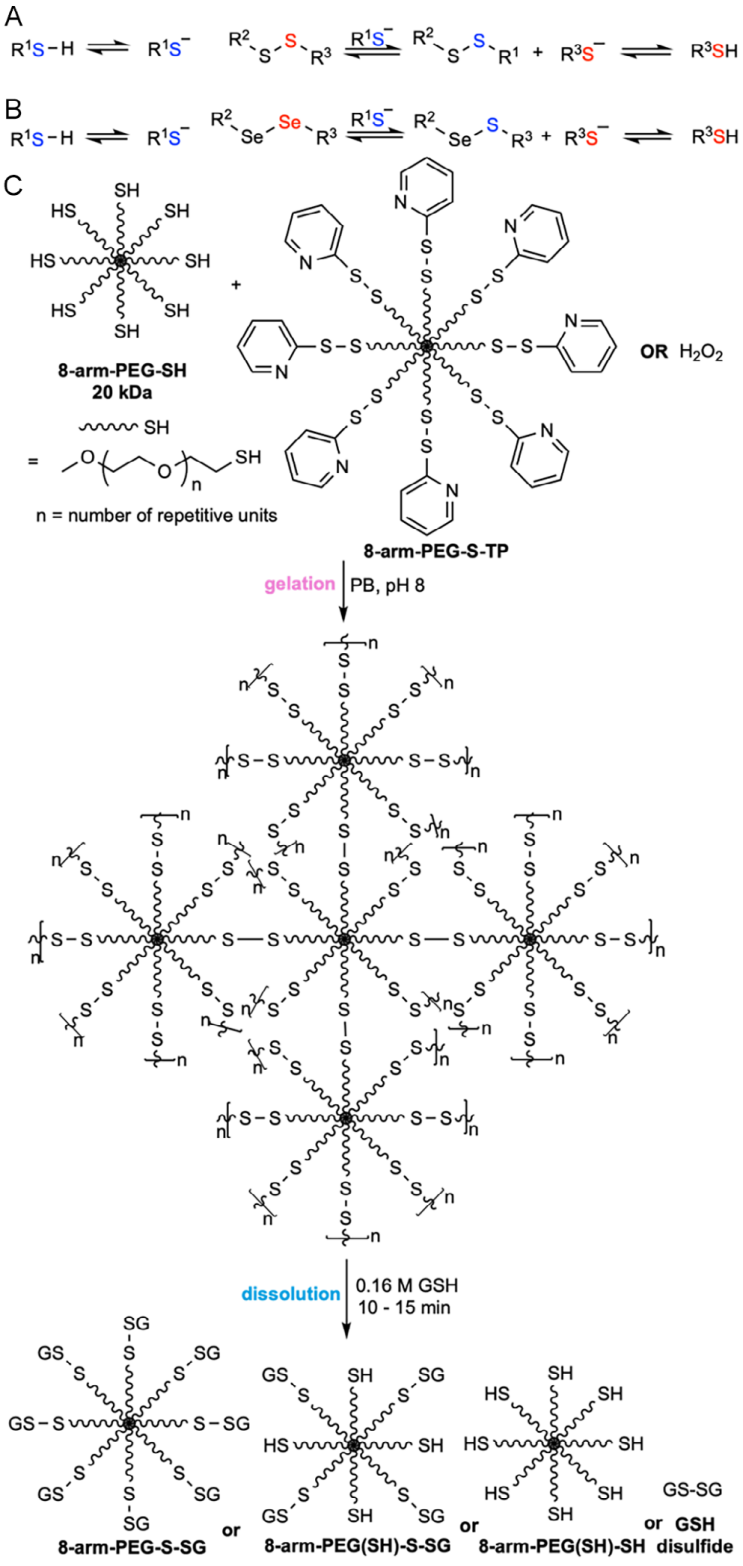

**Figure:**
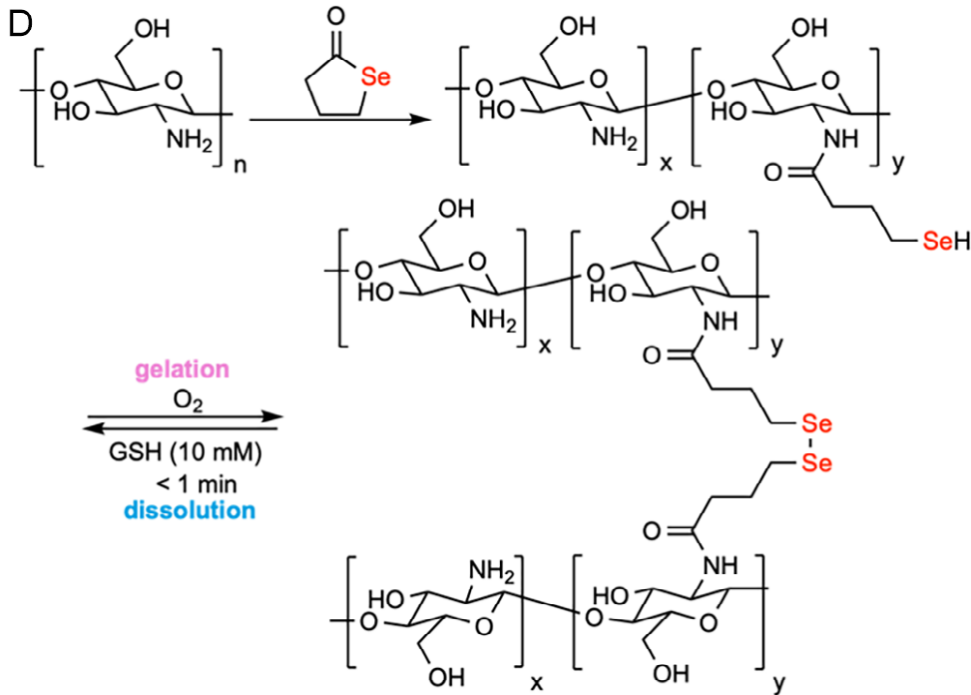

Hydrogels featuring dichalcogenides have been used widely for injectable in situ gelling implants for dermal wound healing [72] or vitreous substitutes [73], as additional small‐molecule crosslinking agents are avoided and the heat of gelation is sufficiently low to avoid damaging surrounding tissues or organs [74]. Notably, in body fluids and extracellular matrices, relatively oxidizing environments exist due to low GSH levels (2–20 µM). Conversely, the intracellular compartment maintains GSH at 0.5–10 mM. Glutathione disulfide is reduced by NADPH‐dependent glutathione reductase, creating a significant redox gradient across the membrane [75]. This difference can be exploited to trigger stimuli‐responsive release of diverse payloads, such as cells [76], proteins [73, 76], siRNA [77], nanoparticles [78], and drug molecules [79]. For example, Sinko and coworkers reported a hydrogel formed in situ by crosslinking an 8‐arm PEG bearing multiple thiols with either hydrogen peroxide or 8‐arm‐PEG‐S‐thiopyridyl (8‐arm‐PEG‐S‐TP). The hydrogel bore high mechanical strength and low swelling (<1.5%) with formulation additives (glycerin, polyvinylpyrrolidone, and PEG‐600) enhancing dermal retention for up to 24 h. In SKH‐1 mice, doxycycline‐loaded (0.25% w/v) hydrogels improved wound healing on nitrogen mustard (NM)‐injured skin versus untreated and placebo controls. Upon exposure to 0.16 M GSH, disulfide crosslinks underwent exchange, and the gels dissolved inducibly in 10–15 min (Figure 3C) [68]. Similar to disulfide crosslinked hydrogels, burn‐dressing hydrogels formed by diselenide crosslinking of *γ*‐selenobutyrolactone‐modified CS demonstrated gel dissolution within 1 min when treated with 10 mM GSH solution, as reported by Xu et al. (Figure 3D) [69].

Hydrogels crosslinked by diselenides and disulfides offer several advantages: gentle gelation without small‐molecule crosslinking agents; injectability and tunability; optical clarity; and removability via competitive thiol concentration or pH adjustments. However, there are some limitations, such as patient‐to‐patient endogenous redox variability, the need for biocompatible oxidants and catalysts for gelation, susceptibility of chalcogenols to air oxidation, necessary routine precautions to avoid premature crosslinking during storage, and the potential to interact with thiol‐bearing molecules or proteins in a biological context. Additionally, selenides have a strong odor and are neurotoxic, which makes them less suitable for various biological applications.

#### 3.1.2 Thiol‐Thioester/Carbonate Exchange

Thiol‐thioester exchange occurs in water under physiological conditions, due to the greater nucleophilicity of sulfur versus oxygen. Mechanistically, the thioester undergoes reversible nucleophilic addition by a thiolate anion in a pH‐dependent manner to form a new thiolate and thioester (Figure 4A). The rate‐determining step depends on the p*K*_a_ of the competing and departing thiols. When p*K*_a‐competing thiol_ > p*K*_a‐departing thiol_, the rate‐determining step is the formation of the tetrahedral intermediate, whereas when p*K*_a‐departing thiol_ < p*K*_a‐departing thiol_, the rate‐determining step shifts to the collapse of the tetrahedral intermediate [83, 84]. L‐cysteine methyl ester (CME) [80, 85, 86], cysteine [87], GSH [87], and water‐soluble 2‐mercaptoethanesulfonate (MESNA) [86] have been reported to cleave the thioester bond. Among these, CME is the most commonly used trigger, proceeding via native chemical ligation (NCL) in which an irreversible intramolecular S → N‐acyl transfer facilitates the exchange process. Notably, the degradation time ranges from 2 min (0.3 M CME in aqueous solution, pH 7.4, r.t.) [85] to 90 min (0.3 M CME in aqueous solution, pH 8.6, r.t.) [81].

FIGURE 4Schematic illustration of the thiol‐thioester/carbonate‐exchange mechanism and examples of on‐demand chemically degradable hydrogels based on this principle. (A) Thiol‐thioester exchange mechanism. (B) Thiol‐carbonate‐exchange mechanism. (C) A burn‐dressing thioester‐crosslinked PEG hydrogel formed from PEG‐bis‐succinimidyl valerate and thioglycolic acid can be degraded when treated with L‐cysteine methyl ester (CME) via thiol‐thioester exchange [80]. (D) A burn‐dressing hydrogel formed between *N*‐hydroxysuccinimide (NHS)‐activated, difunctionalized PEG and amine‐terminated, branched poly(ethyleneimine) (PEI) can be degraded via thiol‐thioester exchange upon spraying with CME [81]. (E) A wound dressing hydrogel crosslinked between aldehyde‐functionalized aliphatic polycarbonate (MBP: MTC‐Ba_*n*_‐*b*‐PEG_4k_‐*b*‐MTC‐Ba_*n*_) and chitosan can be degraded when treated with *N*‐acetylcysteine (NAC) via thiol‐carbonate exchange [82].

**Figure:**
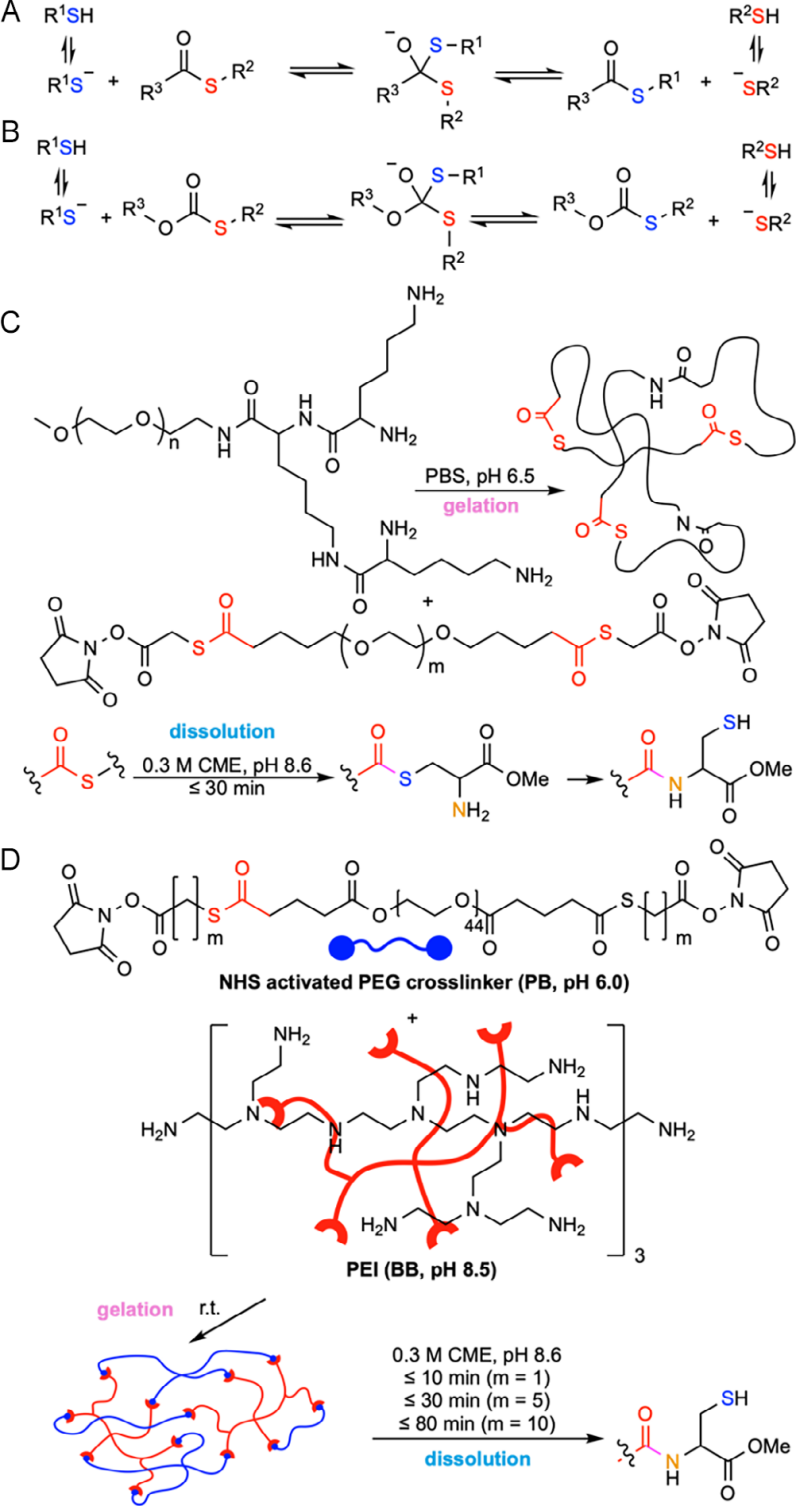

**Figure:**
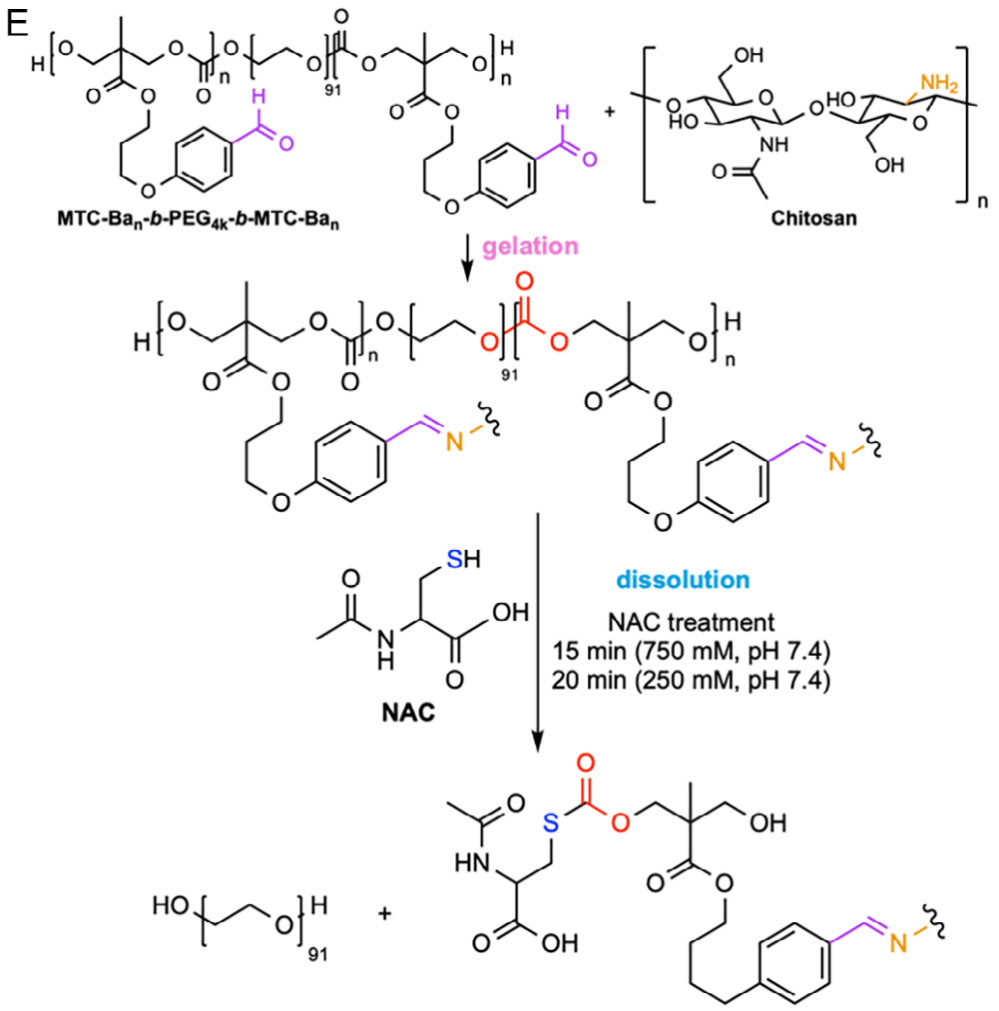

Hydrogels based on this mechanism have been reported to be biocompatible [88], leading to various biological applications including internal wound closure [85], removable dressings for skin burn care [80], cell culture and encapsulation with tunable viscoelasticity [89], chemically triggered drug release [87], and cell–material interaction studies [90]. For example, Grinstaff and coworkers constructed a hydrogel incorporating thioester‐bearing PEG crosslinkers by reacting PEG‐bis‐succinimidyl valerate (SVA‐PEG‐SVA) with thioglycolic acid in the presence of *N*,*N*‐diisopropylethylamine (DIPEA). This hydrogel was used to dress second‐degree burns and to prevent colonization by *Pseudomonas aeruginosa*. When gauze soaked in 0.3 M CME (pH 8.6) was applied to the wound, the gel completely dissolved within 30 min (Figure 4C) [80]. Another injectable hydrogel formed by reacting amine‐terminated branched poly(ethyleneimine) (PEI) with NHS‐activated, difunctionalized PEG, showed the best physical and mechanical properties at 15 wt% for a secondary‐degree burn dressing in in vivo experiments on pig skin. The sample treated with hydrogel showed the least necrosis, epidermal ulceration, and inflammation. When sprayed with 0.3 M CME (pH 8.6), the gel degraded within 10 min (Figure 4D). By comparison, it hydrolyzed over 30 days of swelling [81].

Based on the same principle, on‐demand dissolvable hydrogels that utilize thiol‐carbonate exchange crosslinks have been reported for wound dressing [82]. The dissolution is driven by the associative attack of a thiolate on the carbonate carbonyl, regenerating a free thiol and reshuffling/breaking the network (Figure 4B). For example, Zong et al. reported an injectable, self‐healing hydrogel crosslinked between an aldehyde‐functionalized aliphatic polycarbonate (MBP) and CS through dynamic Schiff base linkages. The carbonate segments in MBP enabled rapid on‐demand degradation triggered by *N*‐acetylcysteine (NAC) via thiol‐carbonate exchange. The optimized formulation (MBP/CS‐gel, 8.4 wt%, 1:1 –CHO/–NH_2_ ratio) formed a stable gel within minutes at room temperature, exhibiting high mechanical integrity and shear‐thinning injectability. Upon exposure to NAC (250–750 mM, pH 7.4), the gel completely dissolved within 15–20 min whereas only slow enzymatic erosion occurred in lipase‐containing PBS, confirming selective thiol‐triggered cleavage of carbonate linkages (Figure 4E). When loaded with 0.2 wt% gallic acid (GA), the composite dressing demonstrated antioxidant and antibacterial activity, excellent cytocompatibility with L929 fibroblasts, and <1.5% hemolysis. In diabetic mouse wound models, GA@MBP/CS‐gels accelerated closure and collagen deposition, achieving nearly full epithelialization by day 20 [82]. This is a significant improvement over standard‐of‐care treatment, in which only about 24% and 31% of uncomplicated ulcers are healed after 12 and 20 weeks, respectively [91, 92].

Thiol‐thioester/carbonate‐exchange hydrogels are known for their tunability, biocompatibility, and chemically triggered removability by competitive thiols; however, cell viability studies have shown that even 5 min exposure of NIH3T3 murine fibroblast cells to 50 mM CME results in just 65% cell viability. Potential metal chelation by CME as well as the hypertonic shock of the high osmolarity solutions employed were cited as potential sources of cytotoxicity [80]. Such concerns are endemic not only to this method but all thiol‐triggered processes, which often rely on high thiol concentrations up to 0.75 M to drive rapid degradation rates [80]. Other concerns that further limit applications include relevant and competitive rates of thioester hydrolysis in water and unwanted cell‐mediated degradation [89].

#### 3.1.3 Retro‐Michael Thiol‐Maleimide (MAL) Exchange

**FIGURE 5:**
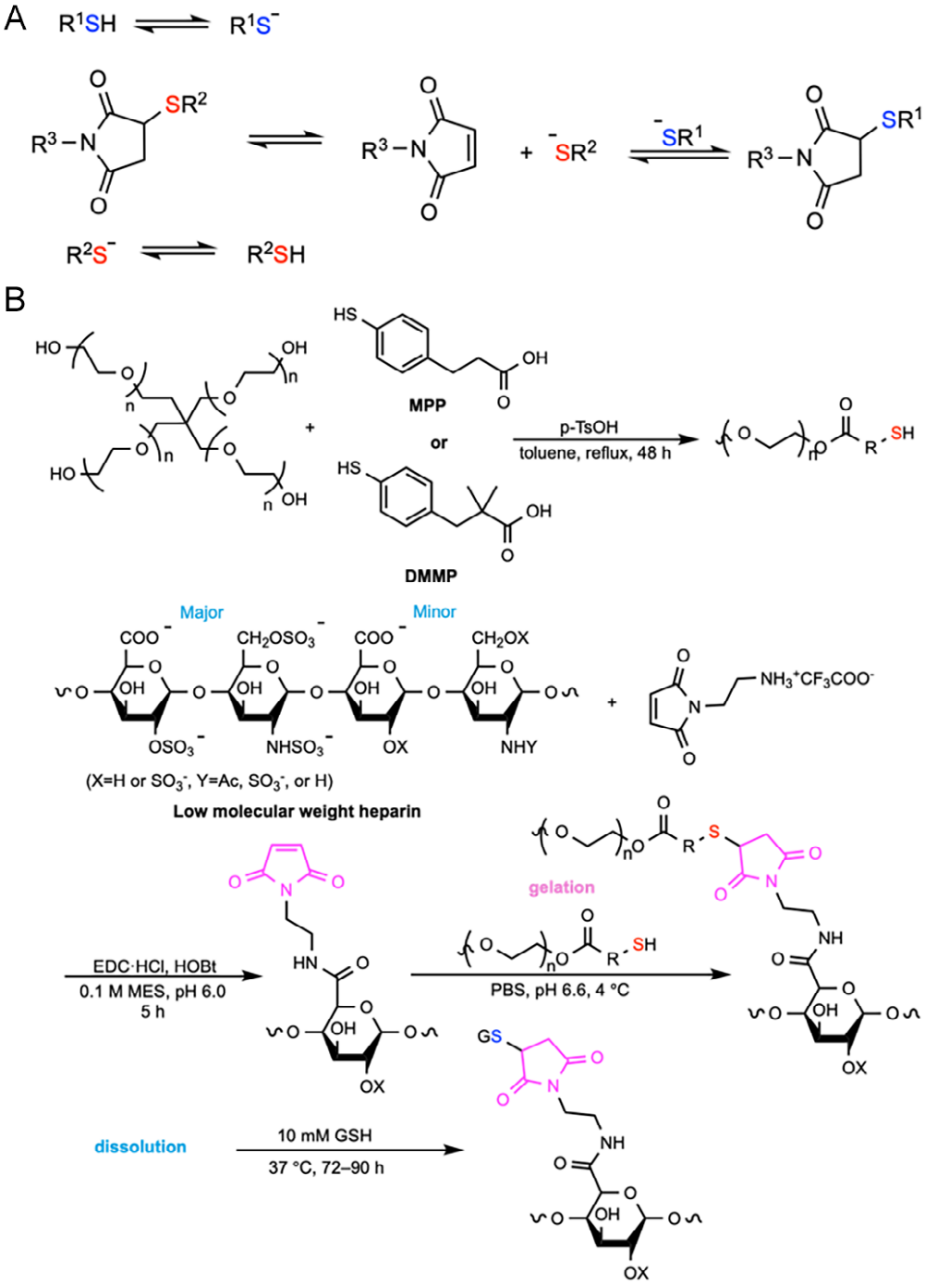
Schematic illustration of the retro‐Michael thiol‐maleimide exchange mechanism and a representative example of chemically induced on‐demand degradable hydrogel based on this principle. (A) Retro‐Michael thiol‐maleimide mechanism. (B) An in situ‐formed hydrogel for heparin release, crosslinked by maleimide‐functionalized low‐molecular‐weight heparin and 4‐arm PEG‐thiols that were functionalized with aryl thiols 4‐mercaptophenylpropanoic acid (MPP) and 2,2‐dimethyl‐3‐(4‐mercaptophenyl)propionic acid (DMMPP), can be degraded when treated with glutathione (GSH) via retro‐Michael thiol‐maleimide mechanism [93].

Thiols add reversibly to maleimides by Michael addition to afford succinimide thioethers. In the presence of competing thiols, the linkage can revert via retro‐Michael exchange, substituting one thiol for another (Figure 5A). It has been reported that the rate of exchange increases with higher thiol nucleophilicity and decreases with steric hindrance. At higher pH (pH = 8), the thiolate fraction rises, accelerating both addition and retro‐Michael exchange, while lower pH (pH = 4) suppresses the exchange [94, 95, 96]. Since amines react at least one order of magnitude slower than thiols, thiol‐maleimide addition is selective at physiological pH, and this mechanism has been applied in developing chemically induced degradable hydrogels [97]. Triggered by competing thiols (e.g., GSH), hydrogels have shown degradation kinetics spanning from hours to days with second order rate constants ranging from 10^–3^ to 10^–4^ M^–1 ^s^–1^ [93]. Thiol‐maleimide hydrogels enable injectable, in situ‐forming depots and tissue‐conformal scaffolds [93, 98] that gel rapidly without catalysts while offering tunable degradation, which is governed by endogenous redox gradients (µM levels of extracellular GSH vs. mM levels of intracellular GSH) or by exogenous thiols for on‐demand removal and controlled release of proteins, drugs, and cells [99].

For example, Kiick and coworkers reported an in situ formed hydrogel that crosslinked maleimide‐functionalized low‐molecular‐weight heparin (MAL‐LMWH) with 4‐arm PEG‐thiols (10 kDa) at 5 wt%, using aryl thiols (4‐mercaptophenylpropanoic acid, MPP, and 2,2‐dimethyl‐3‐(4‐mercaptophenyl)propionic acid, DMMPP) that gel at pH 6.6, 4°C. Their gelation times were within 6 s (MPP) and 15 s (DMMPP), demonstrating injectability. With 10 mM GSH, aryl‐thiol gels dissolved in 72–90 h (Figure 5B). At 10 µM GSH, complete LMWH release required 12 d (MPP) or 20  d (DMMPP) [93]. Further research also demonstrated heparin release as a function of hydrogel dissolution [100].

Advantages of this approach include rapid and selective gelation in water and programmable competitive thiol‐responsive degradability on clinically relevant timescales ranging from days to weeks. However, the exchange rate is slower compared to that of thiol‐disulfide exchange, which is important when rapid clearance is required. Potential maleimide ring‐opening, oxidation or stoichiometric drift of thiols during handling, and site‐to‐site variability stemming from local reductant levels also limit its application.

#### 3.1.4 Diol‐Boronate Ester Exchange

**FIGURE 6:**
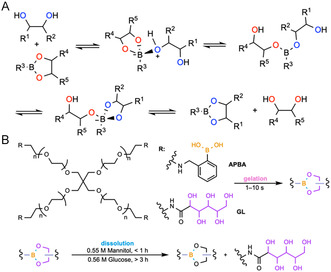
Schematic illustration of the diol‐boronate ester exchange mechanism and a representative example of chemically induced on‐demand degradable hydrogel based on this principle. (A) Diol‐boronate ester exchange mechanism. (B) A hydrogel for thermal protection of biologics crosslinked by four‐arm PEG macromers end‐functionalized with a phenylboronic acid derivative (APBA) and gluconolactone (GL) can be degraded when treated with mannitol or glucose via diol‐boronate ester exchange [101].

Boronate ester hydrogels formed via the reversible condensation reaction between boronic acid and *cis*‐1,2‐ and 1,3‐diols can be degraded by introducing a competing diol molecule to displace the polymer‐bound diol (Figure 6A). The sp^2^ hybridized boron center enforces a 120° O—B—O angle that is mismatched with the optimal geometry of a five‐ or six‐membered diol chelate, introducing angle strain that favors hydrolysis. Formation of the sp^3^ hybridized boron center relieves angle strain, stabilizing the boronate‐diol crosslinks [102, 103]. The esterification is most favored at a pH value both lower than the p*K*_a_ of the diol and higher than that of the acid, as shown in Equation (1) [104, 105]. Deviation from the pH optimal for esterification leads to hydrogel degradation.

(1) $${\mathrm{pH}}_{\text{optimal}}=\frac{pK_{a,\text{acid}}+pK_{a,\text{diol}}}{2}$$

Due to the reversibility of the esterification process and significantly higher concentration of plasma glucose compared to other carbohydrates, boronate ester hydrogel degradation pathways dominated by transesterification through competing diols have been reported [106]. The equilibrium constants for the transesterification of phenylboronic acid (PBA) with diols frequently used in exchange processes span several orders of magnitude depending on the structure of the diol. Quantitatively, *K*_eq,catechol_ (830 M^−1^) is significantly greater than *K*_eq,fructose_ (160 M^−1^) and *K*_eq,glucose_ (4.6 M^–1^) at 25°C in aqueous solutions. On the other hand, linear aliphatic polyols generally exhibit weaker binding with PBA (e.g., *K*_eq,mannitol_ is 120 M^–1^) [107]. Small‐molecule kinetic studies show that boronic acid‐diol exchange is one of the fastest dynamic covalent reactions, with second‐order rate constants ranging from 0.1 to 10^3^ M^–1 ^s^–1^ at room temperature depending on the diol, corresponding to sub‐second to minute‐scale exchange [108]. The hydrolysis of arylboronic pinacol esters at pH 7.4, on the other hand, is relatively slower, with t_1/2_ ranging from 10 min to 3 h [109]. In the context of hydrogels, depending on the pH, binding strengths, and concentrations of these competitive diols, the degradation time ranges from 2 min (15 mM glucose solution, r.t.) [110] to 4 h (25 mM glucose solution, 37°C) [104]. Additionally, the fact that the relaxation time of boronate ester hydrogels does not exceed tens of seconds provides these materials with self‐healing properties, injectability, and motion viscoelasticity [111, 112]. Due to these factors, boronate ester hydrogels have been designed for on‐demand drug‐delivery carriers (e.g., insulin delivery under hyperglycemia conditions through competitive exchange pathways [113, 114], ROS‐responsive immunosuppression [115], or cancer treatment [112] through redox pathways), stimuli‐responsive sacrificial materials for 3D printing [116], cell culture and release [117, 118], and sensors of cell mechanotransduction [119].

For example, Tibbitt and coworkers reported a reversible four‐arm PEG hydrogel that is formed in 1–10 s via dynamic boronic‐ester crosslinking between four‐arm PEG macromers (*M*_*n*_ ≈ 10,000 g mol^−1^) end‐functionalized with a PBA derivative (APBA) and *cis*−1,2‐diol containing moiety [gluconolactone (GL)], which encapsulates biologics for thermal protection and permits on‐demand release with competitive sugars including glucose and mannitol. Mannitol (100 mg·mL^–1^) dissolves the network in <1 h (<10 min when gels are flattened), while glucose requires more time (>3 h) (Figure 6B). Without sugar, dissolution of the gels through hydrolysis requires >24 h. Protein encapsulation preserved >85% *β*‐galactosidase activity after 3 days at 50°C and ≥75% after 4 weeks. The gel protected ≥60% alkaline phosphatase (ALP) activity after 8 days at 65°C and 60% leucine aminopeptidase (LAP) activity after 4 weeks at 50°C. It stabilized clinical enzymes, retaining ≥70% gyrase activity at 4 weeks, ≥50% at 8 weeks, and ≥85% Top1 activity at 6 months, all at 27°C. It also maintained vaccine and virus function, retaining ≥70% H5N1 HA activity at 37°C for 7 days and >90% after 1 day at 60°C, and minimizing Ad5 infectivity losses across 4–65°C with stability maintained for 4 weeks at 27°C in liquid gels. Preliminary in vivo safety experiments showed the nontoxicity of components in rats with renal clearance, supporting translational potential for cold‐chain‐free storage and sugar‐triggered, on‐demand deployment [101].

However, designing these hydrogels at physiological pH remains challenging. Under physiological conditions, most boronate ester hydrogels degrade over hours to days, driven by osmotic forces and changes in phosphate concentration [107, 120], although specially stabilized formulations can persist for weeks to months [121, 122]. Combined with the risk of bacterial proliferation when sugars are added for wound dressing removal and the cytotoxicity and the dose‐dependent cytotoxicity and apoptosis induced by phenol in the low‐millimolar range [123, 124], these factors indicate that boronate ester hydrogels lack bioorthogonality, present design challenges, and are suboptimal for many biological applications.

#### 3.1.5 Amine‐Schiff Base Exchange

**FIGURE 7:**
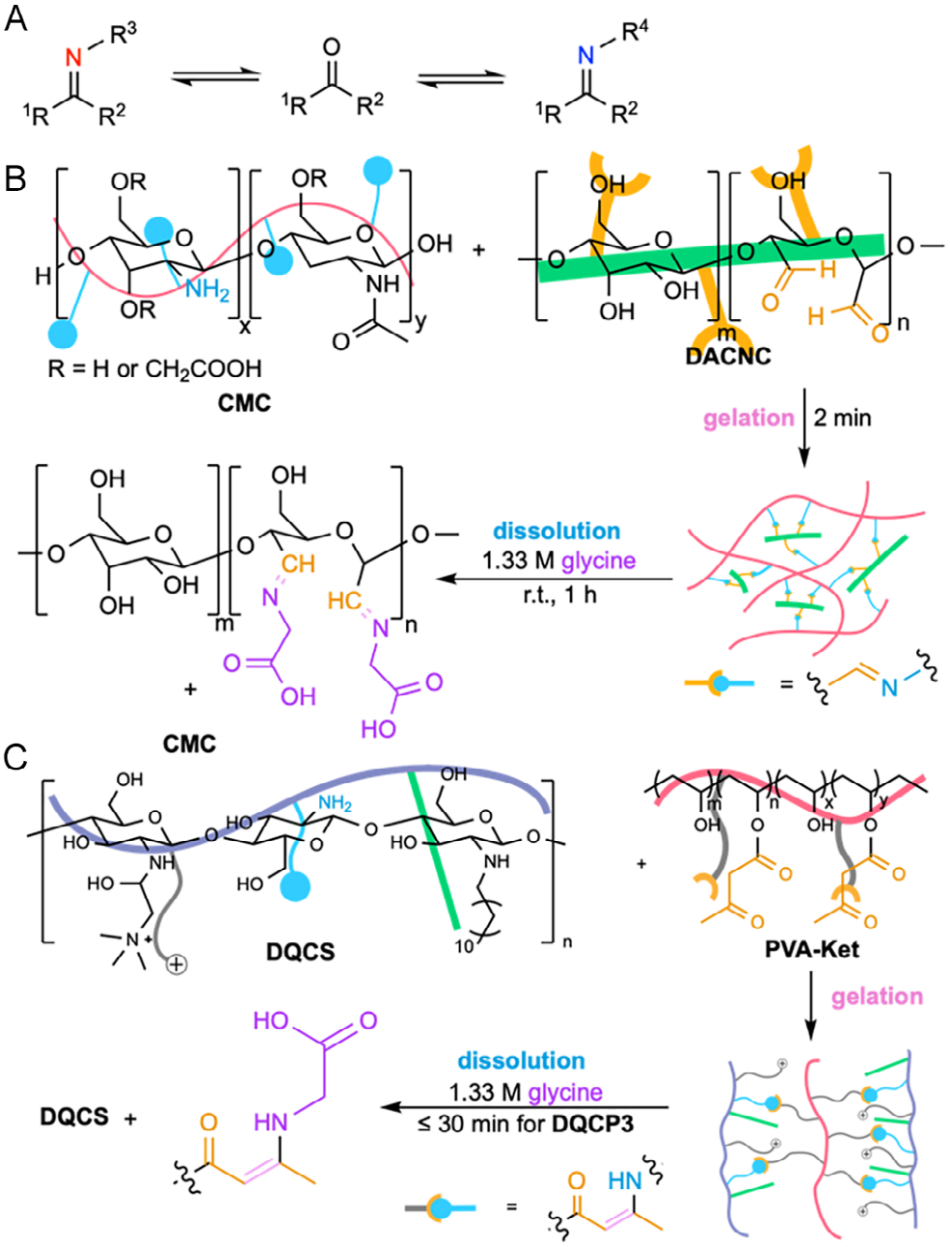
Schematic illustration of the amine‐Schiff base exchange mechanism and examples of chemically induced on‐demand degradable hydrogels based on this principle. (A) Amine‐Schiff base exchange mechanism. (B) A burn‐dressing hydrogel crosslinked by carboxymethyl chitosan (CMC) and rigid rod‐like dialdehyde‐modified cellulose nanocrystals (DACNC) can be degraded when treated with aqueous glucine via amine‐Schiff base exchange [125]. (C) An anti‐inflammatory wound dressing hydrogel (e.g., DQCP3) formed using dodecyl‐grafted quaternized chitosan (DQCS) and ketone‐functionalized PVA (PVA‐Ket) can be degraded via amine‐Schiff base exchange upon incubation with glycine solution [126].

Schiff bases are formed through pH‐dependent dynamic reversible condensation between amines and carbonyl groups. The resulting products undergo hydrolysis in the presence of water and transimination in the presence of competitive amines (Figure 7A). At neutral and alkaline pH, dehydration of the carbinolamine intermediate is generally the rate‐limiting step, whereas at acidic pH, the initial nucleophilic attack of the amine on the protonated carbonyl becomes the rate‐limiting step [127]. Imine hydrogels are known for their biocompatible, self‐healing, and stimuli‐responsive properties. They have been applied to injectable cell encapsulation and delivery [128], wound healing [129, 130], and controlled drug delivery [131]. Common degradation‐inducing amine triggers such as cysteine and glycine induce on‐demand dissolution on timescales ranging from 7 min (0.2 M cysteine, pH 7.4, 37°C) [38] to 1 h (1.33 M glycine solution, pH 6.3, r.t.) [125]. For example, Huang et al. designed an on‐demand dissolvable self‐healing hydrogel for burn wound applications using water‐soluble carboxymethyl chitosan (CMC) and rigid rod‐like dialdehyde‐modified cellulose nanocrystals (DACNC). The gelation happened in situ within 2 min and was perfectly adapted to the wound shape. The gel was completely dissolved after 1 h of incubation in a 1.33 M glycine aqueous solution at room temperature (Figure 7B) [125]. Notably, *α*‐hydrogen‐containing imine bonds can tautomerize to form enamines, which also act as amine‐responsive motifs. Enamine hydrogels have been used for long‐lasting drug delivery [132], wound dressings [126], and 3D‐printing bioinks [133]. For example, Zeng et al. formulated an on‐demand dissolvable, self‐healing enamine‐crosslinked anti‐inflammatory hydrogel by mixing dodecyl‐grafted quaternized CS (DQCS) with ketone‐functionalized PVA (PVA‐Ket) for wound closure. Gel DQCP3 formed in situ in 224 s and dissolved completely within 30 min upon incubation in 1.33 M glycine solution, enabling painless removal from mouse skin (Figure 7C) [126].

Despite their potential, Schiff base hydrogels are susceptible to hydrolysis, particularly in acidic environments, over timeframes ranging from hours to days. Their lack of bioorthogonality is evident in observations of endogenous nucleophiles (protein lysines, N‐termini, small‐molecule amines) triggering degradation and the interaction between residual aldehydes/ketones and proteins leading to irritation or cytotoxicity. These limitations restrict their biological application in long‐term implants, load‐bearing tissue adhesives, cartilage/meniscus scaffolds, chronic drug reservoirs, and wound dressings.

#### 3.1.6 Hydroxylamine‐Hydrazone Exchange

**FIGURE 8:**
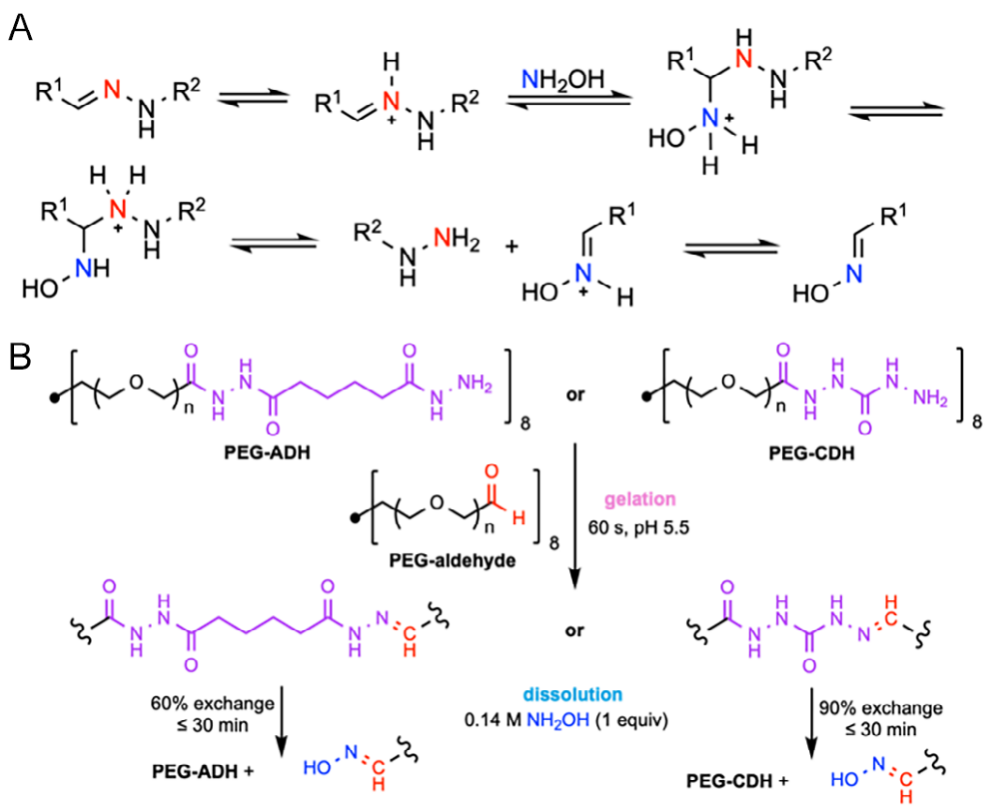
Schematic illustration of hydroxylamine‐hydrazone exchange mechanism and a representative example of chemically induced on‐demand degradable hydrogels based on this principle. (A) Hydroxylamine‐hydrazone exchange mechanism. (B) A hydrogel for tissue engineering crosslinked by reacting PEG‐carbodihydrazide hydrazones (PEG‐CDH) or PEG‐adipohydrazide hydrazones (PEG‐ADH) with PEG‐aldehyde can be degraded via hydroxylamine‐hydrazone exchange when treated with hydroxylamine [134].

Hydrazine or hydrazine‐based nucleophiles (hydrazide/semicarbazide) undergo reversible condensation with aldehydes or ketones to form hydrazones or their derivatives (acylhydrazone/semicarbazone) with water as the only byproduct (Figure 8A). Due to electron delocalization, hydrazones and oximes exhibit better hydrolytic stability compared to the Schiff base [135, 136]. Even though both hydrazones and oximes undergo hydrolysis and nucleophile‐promoted exchange, the degradation of oximes does not occur readily at pH values suitable for cell culture [134]. In biological contexts, hydrazone‐based hydrogels have been used for injectable protein and cell delivery [137, 138, 139], cartilage tissue engineering and regeneration [140, 141], wound healing [142], 3D bioprinting [143], and immunosuppressive tissue adhesive [144]. For example, Boehnke et al. engineered PEG‐based hydrazone/oxime hydrogels for tissue engineering by reacting PEG‐hydrazide with PEG‐aldehyde. Gels formed rapidly in aqueous buffer (≤5 min across pH 5–7, 60 s at pH 5.5) and supported cell encapsulation. Upon addition of 0.14 M hydroxylamine (1 equivalent per hydrazone bond) to hydrogels crosslinked with either carbodihydrazide hydrazones or adipohydrazide hydrazones at room temperature, ≈60% of the carbodihydrazide hydrazone linkages and 90% of the adipohydrazide hydrazone linkages were converted to oximes within 30 min, establishing a practical route to on‐demand degradation of hydrazone networks (Figure 8B) [134].

Hydrazone‐based hydrogels exhibit greater hydrolytic stability compared to Schiff base hydrogels under physiological conditions; however, their rapid gelation rates pose challenges for injectable applications. In addition, endogenous amines and nucleophiles can facilitate slow exchange, while residual aldehydes/ketones may form cytotoxic protein adducts. Water‐sensitive synthesis steps and the limited availability of hydrazide monomers complicate formulation and scale up. Furthermore, hydroxylamines lack bioorthogonality, as they react with endogenous aldehydes and ketones [145] and can attack thioesters such as acetyl‐CoA [146]. It has also been reported that 1 h exposure of 1 mM hydroxylamine to human erythrocytes at 37°C results in significant methemoglobin, glutathione depletion, and lipid peroxidation. Overt hematotoxicity and hemolysis become particularly evident at concentrations of 2.5 mM or higher, which are well below the concentrations used to degrade hydrogels within reasonable timescales. Chronic exposure to hydroxylamine salts has been associated with hematotoxicity, methemoglobinemia, and potential carcinogenic effects [147].

#### 3.1.7 Knoevenagel Condensation C=C Cleavage

**FIGURE 9:**
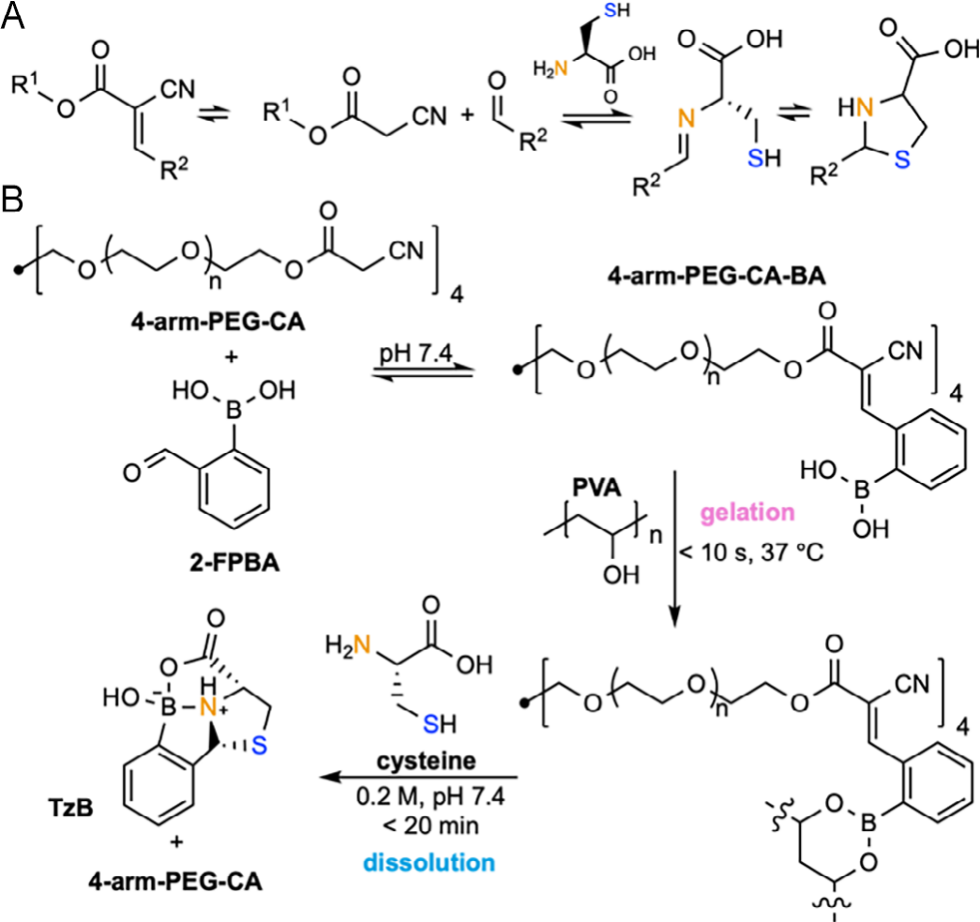
Schematic illustration of Knoevenagel condensation C=C cleavage mechanism and a representative example of chemically induced on‐demand degradable hydrogels based on this principle. (A) Knoevenagel condensation product C=C cleavage mechanism. (B) An in situ‐formed wound healing hydrogel, formed by first reacting cyanoacetate‐terminated 4‐arm PEG (4‐arm PEG‐CA) with 2‐formylphenylboronic acid (2‐FPBA) to produce 4‐arm PEG‐CA‐BA, then crosslinking with polyvinylalcohol (PVA), can be degraded when treated with cysteine via Knoevenagel condensation product C=C cleavage [38].

Knoevenagel condensation describes the coupling of aldehydes or ketones with activated methylene donors. These adducts undergo temperature‐dependent reversible exchange, are sensitive to amine and thiol nucleophiles at physiological pH [135, 136], and can be cleaved through irreversible trapping of the initial carbonyl species by chelating nucleophiles (Figure 9A). Knoevenagel condensation‐based hydrogels have been used in biological applications such as 3D cell culture [148] and wound healing [149], and the degradation of hydrogels constructed by such means has been demonstrated upon application of cysteine. For example, Chen and coworkers reported an injectable self‐healing Knoevenagel condensation‐based hydrogel for wound dressing, which was formed in situ by mixing cyanoacetate‐terminated 4‐arm PEG (4‐arm PEG‐CA), 2‐formylphenylboronic acid (2‐FPBA), and polyvinylalcohol (PVA, 5 wt%) in PBS (pH 7.4). Formation of network crosslinks by catalyst‐free Knoevenagel condensation and boronate ester formation between 2‐FPBA and PVA‐based diols resulted in rapid gelation (<10 s) at 37°C. The Knoevenagel condensation‐derived crosslinks could be undone by exposure to cysteine (0.2 M, pH 7.4). On‐demand dissolution involves imine formation and intramolecular cyclization of the thiol with the reversibly generated benzaldehyde crosslinker to produce thiazolidinoboronate byproducts in 20 min (Figure 9B) [38]. This mode of degradation could be enhanced in combination with an orthogonal diol‐boronate exchange‐mediated degradation mechanism (vide supra), which took place upon addition of 0.2 M glucose.

Knoevenagel condensation‐based hydrogels offer fast, catalyst‐free gelation in water, self‐healing from dynamic C=C linkages, and on‐demand, amine‐triggered dissolution, enabling painless dressing removal and programmable release on clinically relevant timescales. However, Knoevenagel condensation crosslinks are vulnerable to competitive nucleophiles and pH‐dependent exchange. To achieve chemically on‐demand removability within a reasonable time frame, a high trigger concentration is also required. Gel formulation also relies on auxiliary crosslinks (e.g., boronate esters) for stability and on‐demand degradability, thereby introducing complexity into the design of these hydrogels.

### 3.2 Redox Cleavage

Abnormal microenvironments—infected wounds, inflamed tissue, ischemia, and tumors—exhibit increased levels of reactive oxygen species (ROS), including hydrogen peroxide, superoxide, hydroxyl radicals, and peroxynitrite, compared to healthy tissue. Chemical groups, such as thioethers [150], thioketals [151], disulfides [152], vinyldithioethers [153], acryoxalates [154], diselenides [155], monoselenides [156], tellurides [157], arylboronic esters [158], poly(L‐proline) [159], and ferrocene [160], have shown ROS sensitivity, and some of them have been incorporated into stimuli‐responsive hydrogels with demonstrated biocompatibility for wound dressings and drug‐delivery applications. These hydrogels are biodegradable, and it is assumed that they can be degraded by introducing chemical triggers on demand.

#### 3.2.1 ROS‐Thioketal(TK) Redox Cleavage

**FIGURE 10:**
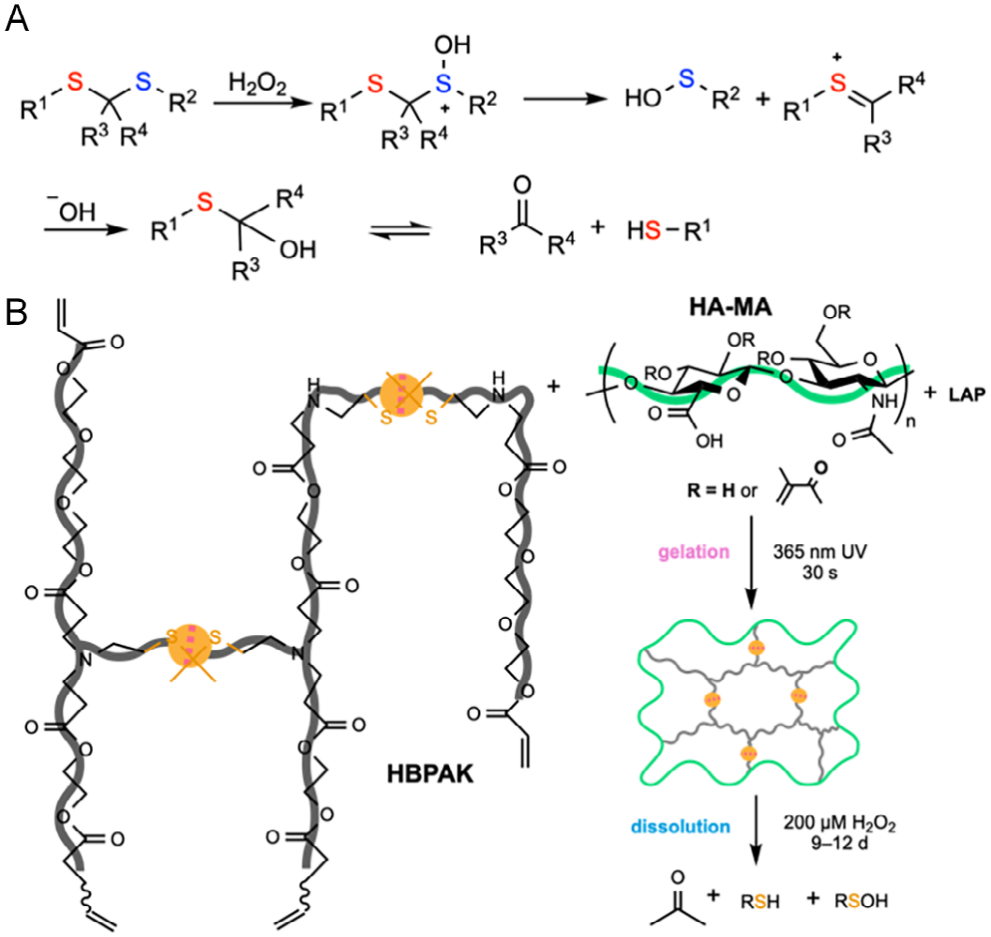
Schematic illustration of ROS‐thioketal redox cleavage mechanism and a representative example of chemically induced on‐demand degradable hydrogels based on this principle. (A) ROS‐thioketal redox cleavage mechanism. (B) A photogelling hydrogel for spinal cord injury treatment, formed between methacrylated HA (HA‐MA) and hyperbranched thioketal‐PEG amine (HBPAK), can be degraded upon treatment with H_2_O_2_ via ROS‐thioketal redox cleavage [151].

In the presence of ROS, thioketals can be cleaved off to form the parent carbonyl through rate‐limiting formation of a thiocarbenium intermediate. The nascent thiol and sulfenic acid can further react to form a disulfide (Figure 10A) [161]. By taking advantage of this chemistry, thioketal hydrogels have been reported to be useful in addressing skin wound healing [162], cell delivery and tissue regeneration [151], stimulus‐triggered drug release [163], and infection control [164]. The degradation kinetics for ROS‐induced degradation span days to weeks, depending on the ROS concentration [165]. For example, Li et al. engineered a ROS‐responsive, thioketal hydrogel for spinal cord injury by photogelling methacrylated HA (HA‐MA, 1 wt%) with a hyperbranched TK‐PEG amine (HBPAK, 10  wt%) and lithium phenyl‐2,4,6‐trimethylbenzoylphosphinate (LAP) using 365 nm UV (30 s; 50 mW cm^−2^). The gel cleared nearly all H_2_O_2_ and 60% superoxide in vitro and degraded much faster under oxidants (51% mass loss at 7 days in 200 µM H_2_O_2_; complete solubilization by days 9–12) than in PBS alone (35% at 7 days; 45% at day 15) (Figure 10B). In vivo, the bone marrow‐derived mesenchymal stem cell (BMSC)‐laden thioketal gel reduced dihydroethidium (DHE)‐detected ROS and anti‐8‐hydroxy‐2‐deoxyguanosine (8‐OhdG) damage markers, shifted macrophages toward proregenerative phenotypes, lowered IL‐1*β*/IL‐6/TNF‐*α*, limited scarring, and improved functional recovery [151].

Advantages of this approach include endogenous ROS sensing and scavenging, with on‐demand, oxidant‐accelerated network dissolution that can be tuned by thioketal density, construct geometry, and local oxidant flux. However, at physiologically modest ROS levels, degradation is often slow—typically degrading over days. From a bioorthogonality standpoint, scavenging and thioketal scission may perturb native redox signaling rather than acting as a clean, external trigger. Moreover, bench use of exogenous H_2_O_2_ is not translatable in vivo, background hydrolysis can erode trigger specificity, and thioketal cleavage generates small‐molecule byproducts that require assessment for tolerance and clearance.

#### 3.2.2 ROS‐Diselenide Redox Cleavage

**FIGURE 11:**
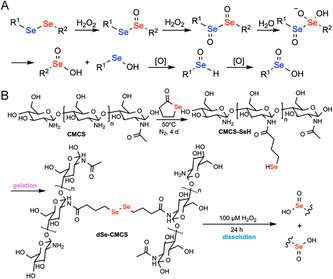
Schematic illustration of ROS‐diselenide redox cleavage mechanism and a representative example of chemically induced on‐demand degradable hydrogels based on this principle. (A) ROS‐diselenide redox cleavage mechanism. (B) An in situ gelling diselenide‐crosslinked carboxymethyl chitosan hydrogel (dSe‐CMCS) for periodontal therapy formed upon air oxidation of selenol‐functionalized CMCS can be degraded upon treatment with H_2_O_2_ via ROS‐diselenide redox cleavage [166].

In addition to being susceptible to competitive exchange, diselenides are also susceptible to ROS‐induced cleavage. Mechanistically, these groups can react with two equivalents of H_2_O_2_ to generate the selenoxide. The doubly oxidized structure hydrolyzes to form the seleninic acid and selenenic acid. The selenenic acid can be further oxidized to form another equivalent of seleninic acid (Figure 11A). In addition to having a weaker Se—Se bond energy compared to disulfides, selenium is more polarizable and easier to oxidize. Autocatalytic pathways can be initiated by the conversion of selenous acid into peroxy acid, which further shifts the equilibrium toward bond cleavage [167]. In contrast, S—S bonds typically result in oxidized sulfoxides or further oxidized sulfones without bond cleavage. On‐demand cleavable diselenide hydrogels exploiting this cleavage mechanism have been widely used for hypoxia‐activated melanoma therapy [168], anti‐inflammation and immune regulation in periodontitis treatment [166], diabetic wound repair [169], and drug delivery [170]. The degradation timescale spans 2 min (2 mL, 3 wt% H_2_O_2_, r.t.) [69] to 4 days (pH 6.8 phosphate buffer + 100 µM H_2_O_2_ at 37°C) [168]. For example, Wu et al. reported an in situ diselenide‐crosslinked CMC hydrogel (dSe‐CMCS) that was loaded with astaxanthin for periodontal therapy. Selenol‐functionalized CMCS (CMCS‐SeH) rapidly gels on air exposure via Se—Se bond formation. These hydrogels could be degraded using 100 µM H_2_O_2_ in PBS, exhibiting progressive structural degradation over 24 h (Figure 11B). In the presence of 100 µM H_2_O_2_, ROS induced 80% release of the colorimetric indicator 1,4‐diamino‐2,3‐dichloroanthraquinone in 72 h compared to ≈40% release observed in the control group [166].

Highly ROS‐sensitive disenlenide‐crosslinked hydrogels allow rapid on‐demand dissolution under near‐neutral conditions and exhibit tunability in crosslink density and geometry to achieve specific combinations of stiffness, degradation rate, and cargo transport and release profiles that match the target tissue and therapy. Moreover, these hydrogels possess ROS scavenging properties, which can contribute to inflammation control. However, the high micromolar concentrations of H_2_O_2_ required for rapid hydrogel degradation pose cytotoxicity issues, considering that normal human plasma contains H_2_O_2_ at concentrations below 50 µM [171]. Furthermore, the consumption of native oxidants reduces bioorthogonality. Cross‐reactivity with selenide and sulfur, air sensitivity, and selenium oxide byproducts also pose handling and safety concerns.

#### 3.2.3 ROS‐Arylboronic Ester (ABE) Redox Cleavage

**FIGURE 12:**
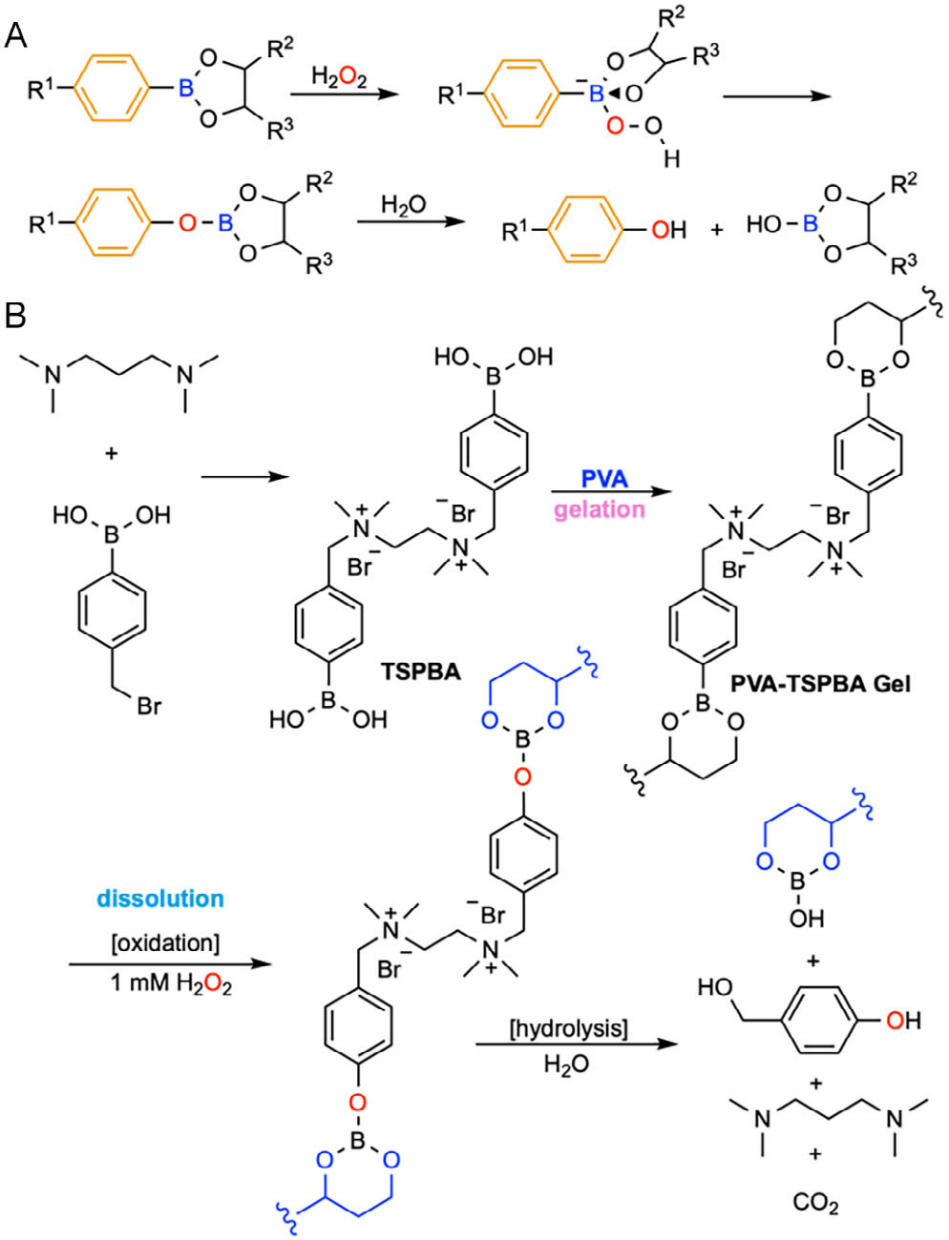
Schematic illustration of ROS‐arylboronic ester redox cleavage mechanism and a representative example of chemically induced on‐demand degradable hydrogels based on this principle. (A) ROS‐arylboronic ester redox cleavage mechanism. (B) An in situ forming ROS‐scavenging PVA‐TSPBA hydrogel for intervertebral disc degeneration, formed by mixing PVA and *N* ^1^‐(4‐boronobenzyl)‐*N* ^3^‐(4‐boronophenyl)‐*N* ^1^,*N* ^1^,*N* ^3^,*N* ^3^‐tetramethylpropane‐1 and 3‐diaminium (TSPBA), can be degraded upon treatment with H_2_O_2_ via ROS‐arylboronic ester redox cleavage [172, 173].

Arylboronic esters can be oxidized by H_2_O_2_ in addition to undergoing competitive exchange. Oxidation begins with a nucleophilic attack on boron to form a peroxyboronate, followed by a 1,2‐aryl migration from boron to oxygen. The resulting intermediate hydrolyzes to phenol and boric acid (Figure 12A) [174]. Notably, this reaction is highly selective for hydrogen peroxide and shows minimal reactivity toward other ROS [175]. On‐demand degradable hydrogels applying this mechanism have been used for injectable depots with ROS‐triggered drug release [176] and sequential antibacterial drug release for wound care [177]. The degradation spans hours [104] to days [176] depending on the trigger concentration. For example, Bai et al. engineered an in situ‐formed ROS‐scavenging PVA‐TSPBA hydrogel for intervertebral disc degeneration by mixing 5 wt% PVA with 5 wt% *N* ^1^‐(4‐boronobenzyl)‐*N* ^3^‐(4‐boronophenyl)‐*N* ^1^,*N* ^1^,*N* ^3^,*N* ^3^‐tetramethylpropane‐1 and 3‐diaminium (TSPBA) and preloading each scaffold with 1 µg rapamycin for local delivery. In PBS at 37°C, gels persisted for more than a week. At 1 mM H_2_O_2_, they progressively degraded over 7 days and released 80% rapamycin within 3 days (Figure 12B). In vivo, photoacoustic imaging confirmed lower H_2_O_2_ levels at the disc, and most gels biodegraded through hydrolysis of the arylboronic ester bond and endogenous diol exchange within 4 weeks in healthy mice [172, 173].

Given that aryl boronic esters are sensitive to diols, pH, and ROS and that the cellular environment comprises both native diols (e.g., glucose, saccharides) and ROS, this mechanism is not considered to be truly bioorthogonal. The reaction kinetics are also slow at physiological ROS levels (10–100 µM H_2_O_2_), often requiring hours to days for complete degradation. At higher (mM) concentrations that induce rapid cleavage, the method risks cytotoxicity. Finally, borate and phenolic byproducts need extra toxicity evaluation to ensure long‐term biocompatibility.

### 3.3 Bioorthogonal Dissociation

Across the major on‐demand chemistries used to dissolve or detach hydrogels, the recurring limitation, especially for clinical use, is imperfect bioorthogonality (Table 1). Competitive thiol exchanges can be tunable but are susceptible to endogenous thiols and protein nucleophiles, often require moderately high exogenous thiol doses, and can drift with local redox/pH, compromising in vivo trigger selectivity. Boronic ester networks are intrinsically sensitive to native saccharides and buffer components, PBA motifs raise cytotoxicity concerns, and achieving stability at pH 7.4 without sacrificing degradability remains challenging. Imine/enamine/hydrazone bonds offer catalyst‐free gelation and exchange, yet residual carbonyls can modify proteins, and endogenous amines might erode crosslinks. ROS‐cleavable motifs sense pathology but consume native oxidants and typically operate slowly at physiological oxidant levels. However, for faster degradation, mM‐scale H_2_O_2_ can be cytotoxic, thereby compromising biocompatibility.

**TABLE 1:** Summary of advantages and limitations of on‐demand chemically degradable hydrogels for biological applications.

Category	Type of interaction		Functional motif	Biocompatible chemical trigger	Advantages	Limitations	Biological applications
Noncovalent disruption	Hydrogen‐bond disruption [45]		Carboxyl‐hydroxyl, carboxyl‐amino, and carboxyl–carboxyl	Chaotropes (urea)	Mild chemistry; reversible thermo‐dissociation; self‐healing	Medium sensitive; pH sensitive; nonbioorthogonal	Hemostasis
	Electrostatic disruption [47]		Carboxylate‐ammonium and sulfate‐ammonium	Salt	Rapid and reversible degradation; easy formulation	Sensitive to ionic strength, pH, and protein content; site‐to‐site variability in vivo; limited to external wounds or surfaces	Wound dressing
	Host–guest complex displacement [48, 52]		Macrocycle host (cyclodextrins, cucurbiturils, crown ethers, pillararenes, and calixarenes) + guest crosslinks	Competitive guest	Injectable; self‐healing	Difficult to establish quantitative structure–property correlations; high local concentration of triggers raise concern for irritation; sensitive to pH, ionic strength, and proteins; host–guest leaching; limited host solubility; high synthesis cost; site‐to‐site variability of endogenous triggers	Burn dressing, wound care, drug depots, tissue engineering
	Metal–ligand coordination disruption [49, 56, 57, 58, 59, 61, 62]		Polymer ligand (catechol, imidazole, carboxylate) + metal ion (Fe^3+^/Fe^2+^, Zn^2+^, Ca^2+^, Mg^2+^, Cu^2+^)	Chelators	Rapid gelation under mild conditions; self‐healing	pH sensitive; ion exchange and competitive ligand exchange in vivo	Bioadhesives, dressings, injectables
Covalent bond cleavage	Competitive exchange	Thiol‐disulfide/diselenide exchange [68, 69, 71, 72, 73, 74, 75, 76, 77, 78, 79]	Dichalcogenide	Competitive thiols (GSH/DTT/cysteine)	Gentle gelation without small‐molecule crosslinking agents; injectable and tunable; optically clear; and removable via competitive thiol concentration or pH adjustments	Local redox variability; need for biocompatible oxidants and catalysts for gelation; sensitivity to air oxidation; precautions necessary to avoid premature crosslinking during storage; thiols potentially interact with thiol‐bearing molecules or proteins in vivo; selenides have strong odor and are neurotoxic	Injectable in situ gelling implants for dermal wound healing or vitreous substitutes, stimuli‐responsive payload release
		Thiol‐thioester/carbonate exchange [80, 81, 82, 85, 86, 87, 88, 89, 90]	Thioester	Competitive thiols (CME/Cys/GSH/MESNA/NAC)	Tunable and biocompatible	High trigger concentration required for rapid degradation is potentially cytotoxic; thioester and carbonate hydrolysis; unwanted cell‐mediated degradation	Internal wound closure, skin burn care, cell culture and encapsulation with tunable viscoelasticity, chemically triggered drug release, cell–material interaction studies
		Retro‐Michael thiol‐maleimide exchange [93, 98, 99]	Succinimide thioether	Competitive thiols (GSH)	Rapid gelation; catalyst free; endogenous redox responsive	Slow competitive thiol‐responsive degradation (days to weeks); maleimide side reactions; local redox variability	Injectable, in situ‐forming depots and tissue‐conformal scaffolds
		Diol–boronate ester exchange [104, 110, 111, 112, 113, 114, 115, 116, 117, 118, 119, 120, 121, 122, 123, 124]	Boronate ester	Competitive diols	Fast dynamic chemistry; self‐healing; injectable	Nonbioorthogonal; design challenge at physiological pH; noninduced degradation over hours to days driven by osmotic forces and changes in phosphate concentration; risk of bacterial proliferation in sugar triggered degradation of wound dressings; dose‐dependent cytotoxicity and apoptosis induced by phenol in the low millimolar range	On‐demand drug‐delivery carriers, stimuli‐responsive sacrificial materials for 3D printing, cell culture and release, sensors for cell mechanotransduction
		Amine–Schiff base exchange [125, 126, 128, 129, 130, 131, 132, 133]	Imine and enamine	Competitive amines (Cys/Gly)	Catalyst‐free gelation; biocompatible; self‐healing	Degradation triggered by endogenous nucleophiles; irritation risks; hydrolysis over hours to days; acid labile	Injectable cell encapsulation and delivery, wound healing, controlled drug delivery, long‐lasting drug delivery, 3D‐printing bioinks
		Hydroxylamine‐hydrazone exchange [134, 135, 136, 137, 138, 139, 140, 141, 142, 143, 144, 145, 146, 147]	Hydrazone	Hydroxylamine	Improved stability compared to Schiff base hydrogels; tunable	Exchange with endogenous amines and nucleophiles; potential cytotoxicity; synthetic and scaling challenge; cytotoxicity of hydroxylamine	Injectable protein and cell delivery, cartilage tissue engineering and regeneration, wound healing, 3D bioprinting, immunosuppressive tissue adhesive
		Knoevenagel C=C cleavage [148, 149]	Knoevenagel adducts	Chelating nucleophiles (Cys)	Fast, catalyst‐free gelation; self‐healing; programmable release on clinically relevant timescales	High trigger concentration; auxiliary crosslinks needed for stability and degradability	3D cell culture, wound healing
	Redox cleavage	ROS‐thioketal cleavage [151, 151, 162, 163, 164, 165]	Thioketal	H_2_O_2_/ROS	Endogenous ROS sensing and scavenging; tunable oxidant‐accelerated network dissolution	Slow degradation at physiological ROS; perturbation of redox signaling; H_2_O_2_ dosing limit; background hydrolysis; thioketal cleavage byproducts require assessment for tolerance and clearance	Skin wound healing, cell delivery and tissue regeneration, stimulus‐triggered drug release, infection control
		ROS‐diselenide cleavage [166, 168, 169, 170]	Diselenide	H_2_O_2_/ROS	Rapid dissolution under near‐neutral conditions; tunable stiffness, degradation rate, and cargo transport and release profiles; ROS scavenging	Perturbation of redox signaling; H_2_O_2_ cytotoxicity; Se safety concerns	Hypoxia‐activated melanoma therapy, anti‐inflammation and immune regulation in periodontitis treatment, diabetic wound repair, drug delivery
		ROS‐arylboronic ester oxidation [104, 176, 177]	Arylboronic ester	H_2_O_2_	Highly selective for H_2_O_2_	Sensitive to diols, pH, and ROS; slow degradation at physiological ROS levels; borate and phenolic byproducts need further toxicity evaluation	Injectable depots with ROS‐triggered drug release, sequential antibacterial drug release for wound care
	Bioorthogonal dissociation	Diboron triggered bioorthogonal dissociation [40]	Enamine *N*‐oxide	B_2_(OH)_4_	Biocompatible, bioorthogonal, rapid on‐demand degradation	Sensitivity to hemeproteins under hypoxic conditions	Wound dressing

**FIGURE 13:**
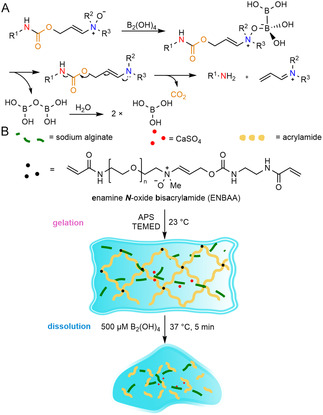
Schematic illustration of bioorthogonal dissociation mechanism and a representative example of chemically induced on‐demand degradable hydrogels based on this principle. (A) Bioorthogonal dissociation mechanism. (B) An oral wound dressing enamine *N*‐oxide hydrogel, formed by mixing enamine *N*‐oxide bisacrylamide (ENBAA), ammonium persulfate (APS), and *N*,*N*,*N’*,*N’*‐tetramethylethylenediamine (TEMED), can be degraded upon treatment with B_2_(OH)_4_ via bioorthogonal dissociation [40].

To address these trade‐offs, a novel chemically on‐demand degradation mechanism was developed by incorporating enamine *N*‐oxides into covalent crosslinkers within robust double‐network hydrogels, enabling rapid and selective bioorthogonal dissociation upon exposure to aqueous tetrahydroxydiboron (B_2_(OH)_4_). Mechanistically, B_2_(OH)_4_ reduces the enamine *N*‐oxide to an enamine, which then undergoes *β*‐elimination of the allylic carbamate to break the crosslink, yielding amine fragments and CO_2_ (Figure 13A). The second‐order rate constant for the rate‐limiting N—O bond reduction is 2 × 10^3^ M^–1^ s^–1^ on small molecules [178] and 82 M^–1 ^s^–1^ on larger macromolecules like proteins [179]. In gels, this translates to dissolution on the timescale of a few minutes. In double‐network polyacrylamide/alginate (PAAm/Alg) hydrogels, which were prepared with 2.2 wt% alginate in Hank's Balanced Salt Solution (HBSS) with acrylamide, ammonium persulfate (APS), *N*,*N*,*N’*,*N’*‐tetramethylethylenediamine (TEMED), and enamine *N*‐oxide bisacrylamide (ENBAA‐1), cast and allowed to fully crosslink for 24 h, exposure to a 500 µM B_2_(OH)_4_ solution in PBS rapidly softened or dissolved the network within 20 min. Partial degradation and near‐complete loss of material performance could be achieved even with exposure to just 100 µM B_2_(OH)_4_. Notably, because of the rapid reaction kinetics, the degradation rates in hydrogels were ultimately limited by reagent diffusion into the hydrogel.

Because double‐network PAAm/Alg swells and loses toughness in wet environments at 37°C, the study also explored thermo‐responsive PNiPAAm/Alg network hydrogels in intraoral wound dressing applications. In ex vivo experiments, 500 µM B_2_(OH)_4_ could be used to successfully remove these gels, which were strongly covalently adhered to pork tongue using CS and EDC/sulfo‐NHS, within 5 min (Figure 13B). Whether delivered by mouthwash (submersion), spray, or solution‐saturated gauze at both r.t. and 37°C, diboron solution could erode adhesion energies measured up to 710 J·m^–2^. Notably, these double‐network hydrogels could be adhered to mice skin and then detached with diboron solution without resulting in any damage to the outer layer of epithelium, as observed by H&E staining. Cell viability studies performed on L929 cells using 500 µM B_2_(OH)_4_ or the hydrogel and its degradation products did not reveal any cytotoxicity. Furthermore, the IC_50_ of B_2_(OH)_4_ is >20 mM for cellular exposure of 30 min and >9 mM for a 3 h exposure. Impressively, mice administered with diboron solution containing up to 150 mg/kg B_2_(OH)_4_ by oral gavage did not exhibit deviations in weight or survival. Both B_2_(OH)_4_ and the boric acid were used or generated, respectively, at concentrations well below reported cytotoxic ranges, demonstrating good biocompatibility [40].

Overall, the degradation rates depended on gel thickness, crosslinker length, and the concentration of B_2_(OH)_4_. The crosslinking modality is very general. Beyond the primary carbamate, alternative linkages, including secondary carbamates, esters, phenyl esters, aliphatic esters, and imides, may be used [179].

In conclusion, enamine‐*N*‐oxide‐crosslinked tough hydrogels enable biocompatible, bioorthogonal, rapid on‐demand degradation with minute‐scale kinetics using aqueous B_2_(OH)_4_ at micromolar concentrations while preserving the favorable mechanical properties of their nondegradable counterparts. A diffusible trigger such as tetrahydroxydiboron is especially advantageous for the uniform coverage of gels with large, amorphous, or irregular geometries.

## 4 Summary and Outlook

On‐demand chemically degradable hydrogels, whether through covalent bond cleavage or noncovalent disruption, offer a practical path to painless dressing removal and traceless dissolution across wound care, cell culture, device interfaces, and drug depots. Compared with nonchemical triggers (e.g., light, heat, ultrasound, magnetothermal), the selling points are inexpensive and scalable trigger reagents, programmable degradation kinetics, simple and uniform degradation in large areas (e.g., spray, rinse, wipe), and the potential for high bioorthogonality with well‐chosen small molecules or chelators. While there are challenges to reagent diffusion in thick, tough gels, bioorthogonal degradation methods offer the best compromise between degradation rate, reagent concentration, biocompatibility, and spatial and temporal precision.

In this review, we framed these materials around stimuli‐responsive mechanisms and removal kinetics. Noncovalent systems favor rapid, gentle detachment using familiar agents at the cost of medium sensitivity. Covalent systems enable decisive, composition‐encoded dissolution and can achieve high bioorthogonality with carefully selected reagents. Properly matched to indication and anatomy, both classes enable easy peel‐off or bulk dissolution after function, reducing pain, inflammation, and procedure time. Looking ahead, the most impactful designs will couple faster kinetics, strong biocompatibility, minimal invasiveness, broad accessibility, and tunable scope of degradation—ideally through hybrid networks that combine a tough, load‐bearing framework with bioorthogonally cleavable bonds or removable supramolecular layers to meet diverse clinical needs.

## Funding

This study was supported by the National Science Foundation (2503344, 2238040).

## Conflicts of Interest

The authors declare no conflicts of interest.
